# Atomic-scale engineering of indium oxide promotion by palladium for methanol production via CO_2_ hydrogenation

**DOI:** 10.1038/s41467-019-11349-9

**Published:** 2019-07-29

**Authors:** Matthias S. Frei, Cecilia Mondelli, Rodrigo García-Muelas, Klara S. Kley, Begoña Puértolas, Núria López, Olga V. Safonova, Joseph A. Stewart, Daniel Curulla Ferré, Javier Pérez-Ramírez

**Affiliations:** 1 Institute for Chemical and Bioengineering, Department of Chemistry and Applied Biosciences, ETH Zurich, Vladimir-Prelog-Weg 1, 8093 Zurich, Switzerland; 2 Institute of Chemical Research of Catalonia (ICIQ), The Barcelona Institute of Science and Technology, Av. Països Catalans 16, 43007 Tarragona, Spain; 3 Paul Scherrer Institute, Forschungsstrasse 111, 5232 Villigen, Switzerland; 4 Total Research & Technology Feluy, Zone Industrielle Feluy C, 7181 Seneffe, Belgium

**Keywords:** Heterogeneous catalysis, Chemical engineering

## Abstract

Metal promotion is broadly applied to enhance the performance of heterogeneous catalysts to fulfill industrial requirements. Still, generating and quantifying the effect of the promoter speciation that exclusively introduces desired properties and ensures proximity to or accommodation within the active site and durability upon reaction is very challenging. Recently, In_2_O_3_ was discovered as a highly selective and stable catalyst for green methanol production from CO_2_. Activity boosting by promotion with palladium, an efficient H_2_-splitter, was partially successful since palladium nanoparticles mediate the parasitic reverse water–gas shift reaction, reducing selectivity, and sinter or alloy with indium, limiting metal utilization and robustness. Here, we show that the precise palladium atoms architecture reached by controlled co-precipitation eliminates these limitations. Palladium atoms replacing indium atoms in the active In_3_O_5_ ensemble attract additional palladium atoms deposited onto the surface forming low-nuclearity clusters, which foster H_2_ activation and remain unaltered, enabling record productivities for 500 h.

## Abstract (web summary)

Generating and quantifying the effect of the promoter speciation in heterogeneous catalysts is very challenging. Here, the authors show that the precise palladium atoms architecture reached by controlled co-precipitation overcomes selectivity and stability limitations associated with palladium nanoparticles for CO_2_-based methanol synthesis.

## Introduction

Metal promotion is a key strategy in heterogeneous catalysis to enhance activity, selectivity, and stability of catalytic centers by tuning of their electronic and geometric properties. Numerous industrial catalysts rely on promoters to comply with requirements related to reactor scale, operating conditions, and atom economy, in order to minimize costs, waste production, and energy input, ensuring higher profits and environmental friendliness^1^. To maximize benefits, the added element should carry the required functionality without introducing detrimental properties, be located in high vicinity to or within the active sites, and remain unperturbed upon use in the targeted reaction. Fulfilling all these goals is a demanding task that necessitates precision synthesis methods defined based on information provided by in-depth characterization, mechanistic elucidation, and testing about the most suitable promoter speciation^2–4^. The latter is a broad-term embracing identity, oxidation state, and molecular structure of the chemical species.

In the frame of research efforts devoted to mitigating global warming and overcoming our reliance on fossil feedstocks^5–16^, promotion by palladium has been attempted on a breakthrough catalyst, In_2_O_3_, recently discovered for CO_2_ hydrogenation to methanol^17,18^. Indeed, although this reducible oxide is very selective and durable, especially when supported on ZrO_2_, owing to the presence of frustrated Lewis pairs^19,20^ and single-ensembles^21,22^, its H_2_-splitting ability is limited^23^. Palladium was shown to generate bulk intermetallic compounds with indium^24,25^, which were superior to In_2_O_3_ in liquid-phase CO_2_ hydrogenation^26^, but were exclusively active for the competitive reverse water–gas shift (RWGS) reaction when applied in the gas phase as nanoparticles supported on silica^27^. A synthetic method devised to deposit palladium nanoparticles on In_2_O_3_ minimizing alloy formation^28^ led to a system displaying enhanced methanol formation, which was ascribed to the higher availability of activated hydrogen, fostering the desired hydrogenation reaction and vacancy formation on the oxide surface. These contrasting findings highlight that the true relevant speciation and action mechanism of palladium are poorly understood. More importantly, to maximize the promoting effect and thus process-level advantages, the H_2_-splitting ability of palladium should be exploited while minimizing its high efficacy for the RWGS reaction as a stand-alone metallic phase^29–31^. As single palladium atoms supported on carbon nitride are active in alkyne semi-hydrogenation and photocatalytic CO_2_ reduction^32–34^ and possess distinct electronic properties compared to agglomerated palladium, calculations indicate that the (R)WGS reaction on Pd(111) requires at least three palladium atoms^35^, and the selectivity of platinum on MoS_2_ in CO_2_ hydrogenation was dictated by the cluster mono-/binuclearity^36^, we conceived atomic dispersion as a strategy to curtail detrimental effects while preserving beneficial attributes. This approach would simultaneously grant improved metal utilization and robustness, if palladium atoms are well anchored to the In_2_O_3_ structure. Aiming at deriving synthesis–structure–performance relations, we emphasized experimental methods as well as theoretical means, which comprise a prominent tool to uncover the impact of structure on activity and selectivity^37–39^ but have rarely been applied to link preparation to structure.

Here, we introduce a co-precipitation method effective in incorporating isolated palladium atoms in the In_2_O_3_ lattice forming low-nuclearity palladium clusters anchored to the active site, as elucidated by state-of-the-art characterization and density functional theory (DFT) investigations. Kinetic and long-term tests show how this speciation overcomes selectivity and stability limitations associated with palladium nanoparticles, rationalizing the unparalleled performance of this catalyst for CO_2_-based methanol synthesis.

## Results

### Impact of promoter nature and loading, and synthesis method

**Fig. 1:**
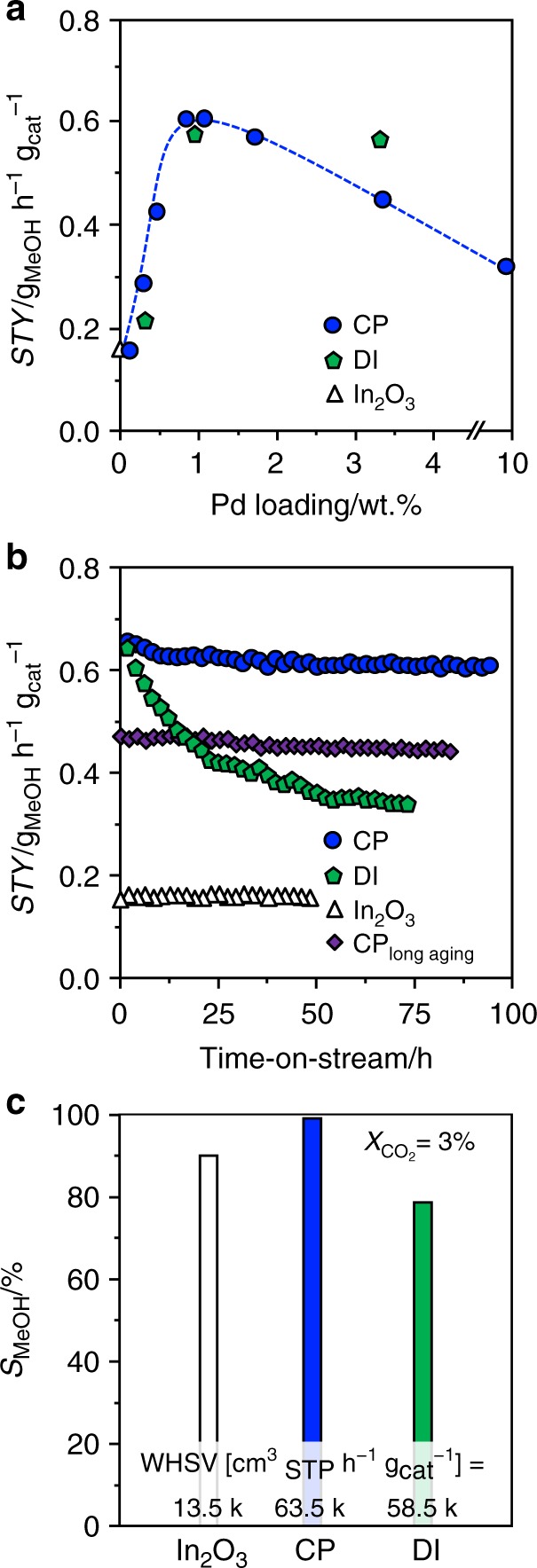
Catalytic performance of palladium-promoted In_2_O_3_ catalysts. **a** Impact of Pd loading on methanol space–time yield (*STY*) after 1 h on stream for palladium-promoted In_2_O_3_ catalysts obtained by co-precipitation (CP) and dry impregnation (DI), **b** temporal evolution of the methanol *STY* for CP and DI catalysts, with pure In_2_O_3_ as a reference, and **c** methanol selectivity over the same materials after 48 h on stream, when a CO_2_ conversion of 3% is attained by operation at the indicated *WHSV*. In **b**, data of an additional CP catalyst is presented that was aged for 200 instead of 1 h. Reaction conditions: *T* = 553 K, *P* = 5 MPa, H_2_:CO_2_ = 4, and *WHSV* = 24,000 cm^3^_STP_ h^−1^ g_cat_^−1^ unless otherwise stated

We prepared palladium-promoted In_2_O_3_ by co-precipitation and a sol–gel method targeting an atomic palladium distribution in the oxide, and applied spray deposition and dry and wet impregnation as well as simultaneous chemical reduction of palladium and indium salts to attain benchmark materials comprising palladium nanoparticles and a PdIn intermetallic compound. All catalysts possessed sufficiently high surface area and pore volume (*S*_BET_ > 100 m^2^ g^−1^, *V*_pore_ > 0.3 cm^3^ g^−1^) and the nominal palladium loading of 0.75 wt.% was closely matched (Supplementary Table 1). Their catalytic performance was assessed in CO_2_ hydrogenation to methanol at 553 K and 5 MPa and compared with that of pure In_2_O_3_. The presence of palladium increased the methanol space–time yield (*STY*) in all cases, except for the intermetallic solid. The co-precipitated catalyst (CP) was the best material (*STY* = 0.61 g_MeOH_ h^−1^ g_cat_^−1^), closely followed by that produced by dry impregnation (DI). Reference solids comprising palladium introduced into the inert TiO_2_ support by CP and DI mainly produced CO, as expected from the reported RWGS activity of palladium nanoparticles^31^. After confirming the superiority of palladium with respect to other typical hydrogenation metals (Supplementary Fig. 1), we unraveled the impact of its loading by testing CP and DI materials containing 0.25–10 wt.% Pd (Fig. 1a). The initial loading of 0.75 wt.% turned out to be optimal, as the *STY* was inferior at lower contents and remained equal or dropped for higher amounts. Interestingly, the CP catalyst with this palladium content retained a *STY* of 0.60 g_MeOH_ h^−1^ g_cat_^−1^ for 93 h on stream after a minimal initial deactivation, whereas the DI counterpart lost half as of its productivity after 74 h (Fig. 1b). Comparing the methanol selectivity (*S*_MeOH_) over CP and DI catalysts and In_2_O_3_ at a CO_2_ conversion of 3% (97, 78, and 89%, respectively, Fig. 1c) revealed that palladium has a beneficial effect on the product distribution when added through CP but not via DI.

### Experimental description of palladium sites

**Fig. 2:**
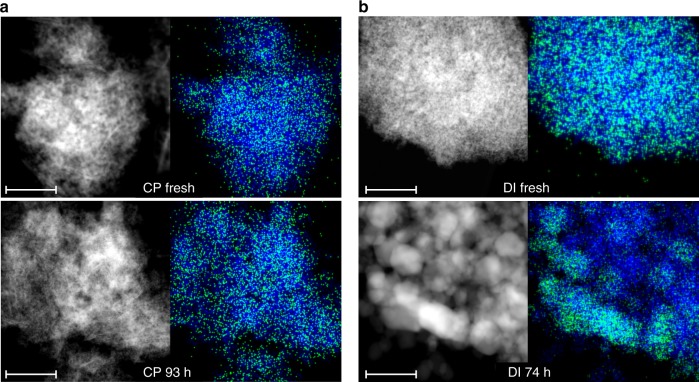
Microscopy analysis of palladium-promoted In_2_O_3_ catalysts. HAADF-STEM images and corresponding EDX maps of indium (blue) and palladium (green) for the catalysts produced by **a** CP and **b** DI with 0.75 wt.% Pd in fresh form and after the tests depicted in Fig. 1b. The scale bar in all panels is 50 nm

CP and DI samples were studied through a battery of characterization techniques to elucidate the palladium speciation. X-ray diffraction (XRD) confirmed the bixbyite (i.e., a defective fluorite-type) structure of In_2_O_3_ in both systems (Supplementary Fig. 2), the lowest-energy surface of which presents a protruding In_3_O_5_(O) substructure, which generates the active site in acetylene and CO_2_ hydrogenation after removal of the (O) atom^21^, and an adjacent sunken region. The average particle size of In_2_O_3_ was calculated at 9 nm for the fresh materials and increased to 15 and 25 nm for used CP and DI catalysts, respectively. Moderate sintering was also observed earlier for unpromoted In_2_O_3_^17,22^. A weak reflection at 39.5° 2*θ* specific to Pd^0^ was detected only for the used DI material. High angle annular dark-field scanning transmission electron microscopy (HAADF-STEM) images and indium and palladium maps acquired by energy-dispersive X-ray spectroscopy (EDX) indicate that palladium is extremely well dispersed in the fresh samples (Fig. 2). The CP material used in the reaction for 93 h appeared virtually unaltered. In contrast, In_2_O_3_ particles increased their size to ca. 30 nm and palladium nanoparticles formed using the DI catalyst for 74 h (Supplementary Fig. 2), the latter having an average size of 2.8 nm based on high resolution transmission electron microscopy (HRTEM, Supplementary Figs. 3 and 4). A determination of the palladium dispersion in CP and DI catalysts was attempted by H_2_ and CO chemisorption. The reduction behavior of the fresh catalysts was studied (Supplementary Fig. 5) through temperature-programmed reduction with H_2_ and CO (H_2_-TPR and CO-TPR) and temperature-programmed desorption of CO (CO-TPD) to identify suitable conditions for the reductive pre-treatment and the volumetric analysis. Barely measurable H_2_ and CO uptakes were registered for used DI samples (Supplementary Fig. 5). We put forward strong metal support interactions (SMSI) and/or Pd–In alloying as plausible reasons for the dramatic change in electronic properties of palladium particles in promoted In_2_O_3_ samples. The similar palladium dispersion determined for a Pd/TiO_2_ material with the same promoter loading and average particle size as DI 74 h (Supplementary Fig. 4), a catalyst for which the SMSI phenomenon has been widely documented^40^, supports our hypothesis. No and a very weak CO chemisorption was detected by diffuse-reflectance infrared Fourier transform spectroscopy (CO-DRIFTS, Supplementary Fig. 6) for fresh and used CP samples and DI 74 h, respectively. The first evidence agrees with studies reporting that isolated charged palladium species do not adsorb CO^32^ and isolated metallic Pd atoms weakly interact with CO at close-to-liquid-nitrogen temperatures^41^, and the second with the volumetric analysis. Based on electron microscopy, atomic dispersion of surface palladium species is plausible for fresh CP and DI catalysts and CP 1 h, while a value of 42% was estimated for DI 74 h considering the TEM-determined average particle size and a spherical geometry^42^.

**Fig. 3:**
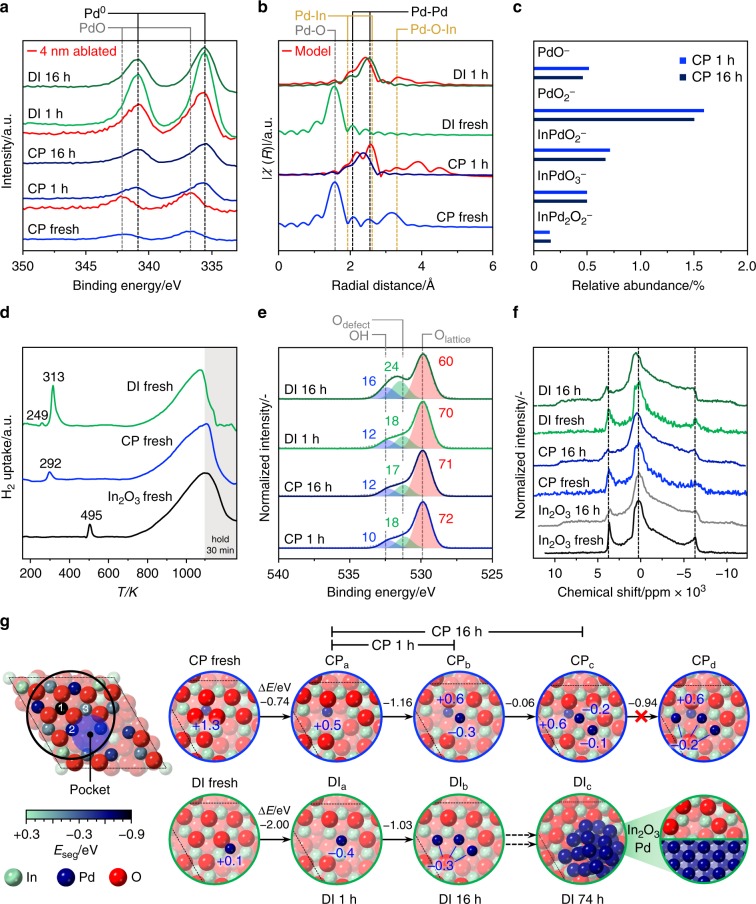
Experimental and theoretical elucidation of palladium-promoted In_2_O_3_ catalysts. **a** XPS Pd3*d* core-level spectra, and **b** Pd–K-edge EXAFS spectra of the CP and DI catalysts in fresh form and after use in CO_2_ hydrogenation for 1 h. EXAFS spectra simulated for the latter are added in red. The dashed lines mark expected positions of signals of the species indicated^52–54^. **c** Abundance of Pd-containing fragments emitted from the surface of used CP and DI materials in TOF-SIMS analysis. **d** H_2_-TPR profiles at 5 MPa of the fresh CP and DI catalysts with In_2_O_3_ as a reference. **e** XPS O1*s* core-level spectra of CP and DI catalysts after use for 1 and 16 h. The contribution of lattice oxygen, oxygen close to a defect, and oxygen in a hydroxyl group retrieved from deconvolution are marked in red, green, and blue, respectively, and labeled with their relative abundance in %. **f** In^115^ NMR spectra collected for pure In_2_O_3_ and the CP and DI catalysts in fresh form and after use for 16 h. **g** (left) Structure of a regularly terminated In_2_O_3_(111) surface featuring, owing to the strong anisotropy, a depression (pocket) where Pd atoms preferentially accommodate in the fresh DI catalyst. The coloring of In atoms at the surface represents the energy associated with their replacement by palladium (*E*_seg_, in eV), which takes place upon CP. 1–3 mark the lattice positions most preferred by the promoter. (right) Initial palladium-promoted In_2_O_3_(111) surface models for the CP and DI catalysts and their evolution to account for structural modifications occurring upon their use for in the reaction. The energy difference between states for each transition (Δ*E*, in eV) and the Bader charges of the Pd atoms (*q*_B_ in |e^‒^|) are indicated in blue. Atoms are shaded with a progressively lighter color upon increasing distance from the surface towards the bulk

According to X-ray photoelectron spectroscopy (XPS), the fresh CP and DI catalysts exclusively contained Pd^2+^ species (Fig. 3a), which chiefly reduced to Pd^0^ upon use in the reaction for 1 h. Residual Pd^2+^ species are likely owing to the short exposure of the samples to air for the ex situ analysis, as no uptake possibly related to the reduction of oxidized palladium was evidenced by H_2_-TPR for CP used for 1 h at H_2_:CO_2_ ratios of 4 and 2 (Supplementary Fig. 7). Despite the same bulk metal loading, palladium is almost 3-times more abundant on the surface of the DI catalyst (1.1 at.%, Supplementary Table 2) than on the CP sample (0.4 at.%), which supports Pd incorporation throughout the oxide crystals in the latter. After removing ca. 4 nm of the surface by sputtering with Ar^+^ ions, metallic palladium was still detected for DI 1 h. As this material only contains palladium deposited onto the In_2_O_3_ surface, the signal of the promoter should vanish. Still, information on the bulk is obviously mixed with that of the surface, as we are dealing with a polycrystalline material with an average particle size of ca. 8 nm, and XPS has a penetration depth of ca. 6 nm. For CP 1 h, palladium in oxidized form was evidenced for the sputtered sample, suggesting that the promoter in the interior of the oxide is not reduced upon reaction. The Pd3*d* signal of CP 16 h exhibited unaltered shape and intensity, as expected based on the stability of this material. In contrast, the surface Pd content for the DI material almost halved after 16 h on stream, in agreement with its sintering.

To further elucidate the palladium speciation in the two systems, X-ray absorption near edge structure (XANES) and extended X-ray absorption fine structure (EXAFS, Fig. 3b) spectroscopy were measured at the Pd K-edge. The fresh CP material contains isolated Pd^2+^ species, which possess four O neighbors (Pd–O scattering path, Supplementary Fig. 8 and Supplementary Table 3) in square geometry at a distance of ca. 2.01 Å in the first coordination shell and are embedded in In_2_O_3_, as evidenced by the second coordination shell of Pd following the Pd–O-In scattering path. Accordingly, they are strongly bound to the oxide surface or can be in bulk lattice positions if oxygen vacancies are present in their first coordination shell. For the fresh DI catalyst, the O shell around Pd is similar to that in the CP material and in PdO, but the interaction with the In_2_O_3_ lattice is much weaker, as hinted by the low coordination number of the Pd–O-In scattering path. Consequently, palladium is likely present as an oxide highly dispersed on In_2_O_3_. After 1 h on stream, palladium becomes metallic in both catalysts forming nanostructures smaller than in the intermetallic reference, which contains nanoparticles of ca. 2 nm. In the CP material, these actually comprise clusters of  4–5 atoms, which do not grow significantly upon longer use in the reaction. Due to the similar scattering factors of palladium and indium, their composition can be described as Pd_*x*_In_4‒*x*_ tetramers or Pd_*x*+1_In_4‒*x*_ pentamers. The resemblance of the XANES spectrum of the used CP sample to that of the intermetallic reference suggests that these clusters include both elements. As the Pd K-edge was probed, one atom must be Pd. As Pd–O-In scattering is detected and the palladium amount is low, it is unlikely that the remaining 3–4 atoms are all Pd atoms. Considering that palladium modifies the intrinsic activity of the catalytic site in indium oxide, it is reasonable that palladium replaces one of the In atoms in the In_3_O_5_ ensemble. Hence, most likely configurations are Pd_2_In_2_ and Pd_3_In_2_. These species can only be generated by CP and with low palladium loading, as the Pd–O-In scattering is absent in the EXAFS spectra of the CP catalyst with 3.5 wt.% Pd and the DI solid. Confirmation of the low clustering degree of palladium was attained by time of flight-secondary ions mass spectrometry (TOF-SIMS). Ionic species emitted from the surface of CP 1 h predominantly contained a single Pd atom, and no ions containing > 2 Pd atoms. Relevantly, an equivalent result was attained for CP 16 h, in line with the high resilience of palladium to aggregate in the CP catalyst (Fig. 3c).

To investigate the promoter impact on vacancy formation, H_2_-TPR at the process pressure (5 MPa, Fig. 3d and Supplementary Fig. 9) was conducted. Surface reduction of In_2_O_3_ occurred at much lower temperatures for all promoted catalysts than for the unpromoted oxide (292–313 versus 495 K), highlighting the assistance of palladium in H_2_ splitting. The signal of the DI sample is more intense, hinting at a higher number of vacancies. For the same solid, an additional signal at 249 K related to palladium reduction was detected, which supports the segregation of the promoter from In_2_O_3_. The findings are further corroborated by deconvolution of the XPS O1*s* signal (Fig. 3e): CP and DI used for 1 h contain a comparable amount of vacancies and after 16 h on stream their concentration remains practically unaltered for the CP catalyst, whereas it increases by 29% for the DI system. The latter sample also features a greater amount of surface hydroxides (25% more than CP), likely caused by dissociated hydrogen spilled from palladium particles to In_2_O_3_. A greater abundance of vacancies is also supported by the higher quantity of CO_2_ desorbed from this catalyst than from CP 1 h (Supplementary Fig. 10). To further unravel changes of the oxide by palladium addition, ^115^In solid-state nuclear magnetic resonance (NMR) spectra were acquired to selectively probe indium. Notably, the large quadrupolar interaction of the NMR active spin 9/2 In nucleus makes this a challenging analysis, which has never been reported for a bulk indium-based material. Two magnetically inequivalent types of In atoms were detected in the spectrum of cubic In_2_O_3_, one causing the central transition with maximum at 280 ppm and the other originating two peaks at 3750 and ‒6250 ppm (Fig. 3f). The asymmetry of these functions is owing to quadrupolar coupling and some disorder, owing to the small size of the oxide particles. The spectra of fresh CP and DI catalysts resemble that of In_2_O_3_, but are noisier, mostly because of the diamagnetic character of the PdO present. Additional signals between 5000 and 8000 and −7000 and 11,000 ppm were observed for CP 1 h and DI 1 h owing to the formation of oxygen vacancies, which modify the electronic environment of adjacent In nuclei. Although these are equivalent in all used samples, the particles size of In_2_O_3_ in DI 16 h is larger, implying a higher density of vacancies per surface area in this material. The analysis further indicates that the In_2_O_3_ structure must be preserved in models of promoted catalysts selected for theoretical modeling.

### Theoretical description of palladium sites and mechanism

Different palladium-promoted In_2_O_3_(111) surfaces were analyzed to unravel the most likely environment of the noble metal in CP and DI catalysts (Fig. 3g). As palladium and indium cations are atomically mixed upon CP, palladium can replace indium in the In_2_O_3_ lattice. However, DFT simulations indicate that palladium is substantially more stable at a surface rather than a bulk position, i.e., segregation is favored towards position **1** in the In_3_O_5_(O) ensemble (− 0.87 eV). The replacement of a second or third In atom in the ensemble is unfavored, with islanding energies taking positive values (up to 0.75 eV, Supplementary Table 4). The Pd coordination is square planar, with a Pd–O distance of 2.05 Å, in good agreement with the experimental EXAFS data for the fresh sample. The substitution of Pd atoms in the In_3_O_5_(O) protrusion promotes the formation of two oxygen vacancies upon reaction (Supplementary Fig. 11), creating a PdIn_2_O_4_ site. Hence, Pd species become more reduced, as evident from the core-level Pd3*d* spectra and the Bader charges (Fig. 3g). The Pd_*x*_In_4‒*x*_ and Pd_*x*+1_In_4‒*x*_ clusters identified by EXAFS in the used CP sample can be rationalized by the spontaneous migration of Pd atoms from the small depression (pocket) to the adjacent PdIn_2_O_4_ sites. Still, an ensemble with 2 Pd atoms likely is more representative of the actual catalyst than one with 3 Pd atoms, as the fresh catalyst contains only few extra-lattice Pd atoms (Supplementary Table 2), the addition of a Pd atom to a palladium dimer is thermoneutral, and configurational entropy favors dispersion^43^. Notably, the electronic structure of Pd_2_In_4_O_4_ ensembles strongly differs from that of In_2_O_3_-supported Pd nanoparticles^28^ and PdIn intermetallics^27,44^ based on local densities of states calculations (Supplementary Fig. 12). However, CP models with 0–4 Pd atoms were considered for the reaction mechanism study detailed below.

**Fig. 4:**
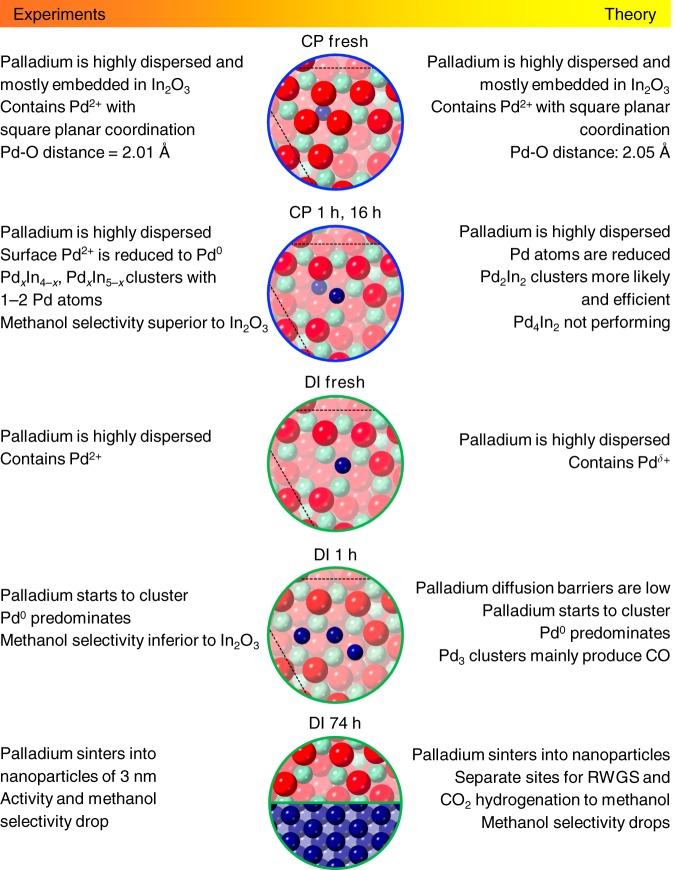
Experimental and theoretical description of palladium sites. Most relevant surface models representing the CP and DI catalyst in fresh, activated, and used forms and their main features derived from experiments and theory. The color code for the atoms in the models is the same as described in caption to Fig. 3

In the case of DI, Pd atoms are deposited onto an already formed oxide surface preferentially allocating in its depressions. Upon exposure to the reaction mixture, the oxidic palladium species lose the ligands becoming metallic and tend to generate nanoparticles since (i) clustering energies are favorable (−0.4 to −1.0 eV), (ii) Pd atoms on the surface are more abundant than in the CP material (Supplementary Table 2), and (iii) the barriers for the diffusion of Pd atoms between pockets are mild (Supplementary Fig. 13). This phase separation leads to a DI catalyst exhibiting In_3_O_5_(O) ensembles and Pd nanoparticles as active sites. The latter easily activate H_2_ and, by spillover, promote the formation of additional vacancies on the oxide surface (Fig. 3g). Simulated EXAFS spectra of the active structures in CP and DI solids are in reasonable agreement with experimental findings (Fig. 3b). Figure 4 presents the most significant CP and DI catalysts’ models and their features, highlighting the remarkable matching of information derived from experiments and from theory.

Gibbs energy profiles for methanol and CO formation from CO_2_ were calculated based on the elementary steps found optimal for In_2_O_3_. The activation of the CP catalyst starts with H_2_ dissociation onto two O atoms located nearby the lattice palladium species, followed by transfer of one H atom to form water, which desorbs creating a vacancy. A second vacancy is generated similarly (Supplementary Fig. 11). This can be followed by the creation of active sites with higher palladium nuclearities, i.e., Pd_*x*_In_2_O_4_ (*x* > 1), through palladium migration from pockets to PdIn_2_O_4_ ensembles. For the DI catalyst, vacancy formation is equivalent, but H_2_ is split on Pd atoms in the pockets. Notably, H_2_ dissociation on In_2_O_3_ is heterolytic, whereas it is homolytic in the presence of intra- or extra-lattice Pd atoms, which reduce the H_2_ splitting barrier. CO_2_ hydrogenation to methanol on CP and DI surface models with 1–4 Pd atoms comprises CO_2_ adsorption (Supplementary Figs. 14–17), followed by H_2_ activation and sequential transfer of the two adsorbed H atoms to the C atom. Further H_2_ activation enables the hydrogenation of one O atom leading to H_2_COOH. Cleavage of the OH group generates a CH_2_O species, which is hydrogenated at the C and the O centers, in this order, producing methanol. The latter then desorbs. In the RWGS reaction, CO_2_ adsorbs on the surface once a third vacancy is created, and is hydrogenated at one O atom. The formed OH group is cleaved and moves to a vacancy, whereas CO desorbs. Based on the span model, CP surfaces with 1–3 Pd atoms and the DI surface with 1 Pd atom are selective towards methanol and to a greater extent than bulk In_2_O_3_, whereas, for the CP surface with 4 Pd atoms and the DI surface with 3 Pd atoms, the intrinsic activity of palladium for the RWGS reaction dominates and CO is the favored product. This agrees with the prerequisite of 3 extra-lattice Pd atoms to mediate the water–gas shift reaction on TiO_2_-supported palladium^35^. The fact that the boost in methanol selectivity is limited for the CP model with a sole palladium species and remarkable for those with 2–3 Pd atoms supports the higher relevance of Pd_2_In_2_O_4_ and Pd_3_In_2_O_4_ as active sites. Regarding the equilibrated DI catalyst, CO_2_ hydrogenation can be described as a superposition of RWGS on palladium, i.e., the Pd(111) surface^35,45^, and methanol and CO formation on neighboring undoped In_2_O_3_^22^, the latter being facilitated by hydrogen spillover from adjacent palladium nanoparticles. To account for coverage effects in this complex surface reaction, a microkinetic model was built (vide infra).

### Experimental selectivity-promoter nuclearity correlation

Intrigued by the selectivity-nuclearity correlation uncovered by the theoretical studies for CP catalysts, we conducted a complementary experiment to link palladium agglomeration and its impact on methanol selectivity. When the DI catalyst was tested keeping the CO_2_ conversion at 2–3% (Supplementary Fig. 18), the methanol selectivity remained at ca. 83% for 6 h, followed by a linear decline owing to a progressively greater impact of the RWGS reaction till a value of 58% after 9 h on stream. Characterization of the specimen retrieved at the end of the test confirmed severe sintering of palladium and In_2_O_3_ (particle sizes of 12 and 28 nm, respectively). A sample generated by a repeated experiment ending before the onset of the selectivity change still showed an extremely high-palladium dispersion based on STEM-EDX, as expected. This supports that agglomerates of only few Pd atoms are required to start favoring the RWGS reaction and highlights the importance of controlling the promoter nanostructure upon synthesis and preserving it upon reaction.

To tackle the former aspect, we explored the impact of the aging time in CP, as continuous dissolution and re-precipitation occur at this stage, which could lead to palladium segregation from In_2_O_3_. For aging times up to 48 h, the methanol selectivity of CP catalysts was unaltered at ca. 80%, hinting that palladium clusters formed still were very small (Supplementary Fig. 19). A sensible difference was observed for the material aged for 200 h (64%), which was found to comprise large palladium particles (up to 10 nm) as well as finely distributed palladium (Supplementary Fig. 20), with its performance originating from the superposition of these two distinct promoter speciations. The In_2_O_3_ particle size was 12 nm, corroborating dynamic restructuring upon aging. The longer term behavior of this sample evidenced less-pronounced deactivation than for the DI catalyst and a sustained methanol *STY* intermediate between that of the CP catalyst attained with an aging of 1 h and the DI solid.

### Evaluation of reaction kinetics and catalyst durability

**Fig. 5:**
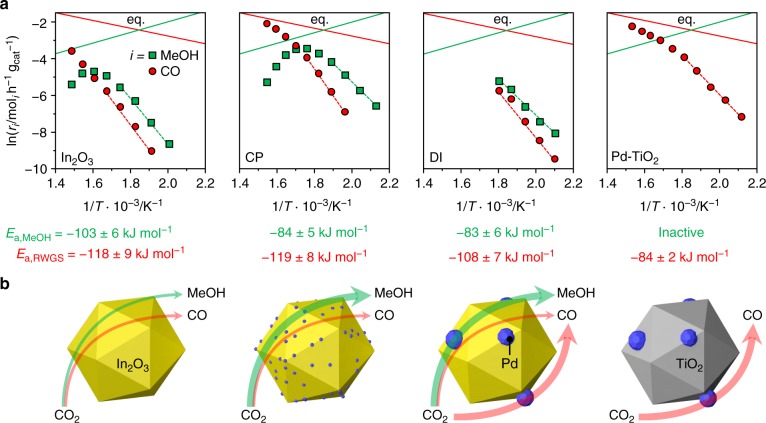
Activation energies and palladium role for palladium-promoted In_2_O_3_ catalysts. **a** Arrhenius plots and calculated apparent activation energies (*E*_a_) for CO_2_ hydrogenation to methanol (green) and the RWGS reaction (red) over In_2_O_3_, CP, DI, and Pd-TiO_2_ catalysts. Data regression was performed in the region outlined by the dashed lines. Solid lines indicate the thermodynamic equilibria of the two independent reactions. **b** Schematic representation of the distinct role of palladium in equilibrated CP and DI systems with In_2_O_3_ and Pd-TiO_2_ as references. The thickness of the arrows qualitatively indicates the products formation rates. Reaction conditions: *P* = 5 MPa, H_2_:CO_2_ = 4, and *WHSV* = 48,000 cm^3^_STP_ h^−1^ g_cat_^−1^

**Fig. 6:**
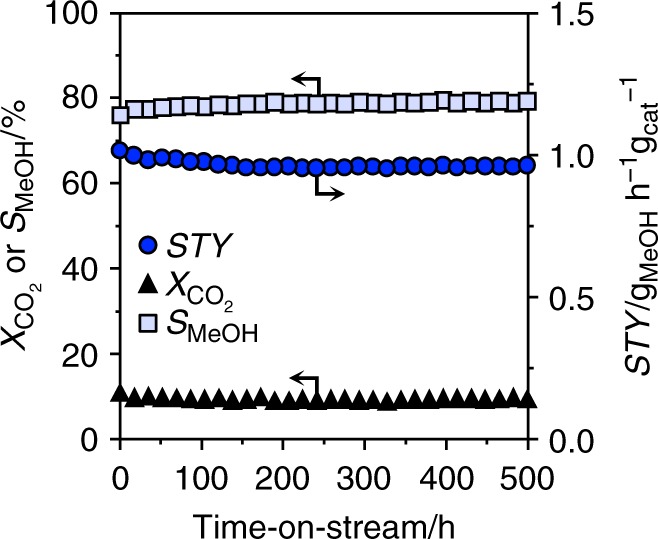
Extended stability test of the best palladium-promoted In_2_O_3_ catalyst. Evolution of the catalytic performance of the CP catalyst during a 500-h test conducted under optimized reaction conditions: *T* = 553 K, *P* = 5 MPa, H_2_:CO_2_ = 4, and *WHSV* = 48,000 cm^3^_STP_ h^−1^ g_cat_^−1^

To gain insights into the origin of palladium promotion, the kinetics of CO_2_ hydrogenation was investigated in detail. An experimental analysis was performed over the CP catalyst, the DI material used for 48 h, and the Pd/TiO_2_ catalyst already serving as a reference for characterization purposes, under the assumption of first order kinetics, as determined for In_2_O_3_^22^. Moreover, a microkinetic model was developed and applied to all CP and DI models^35,45^. Based on experimental tests at variable temperature (Fig. 5a and Supplementary Fig. 21), the apparent activation energy (*E*_a_) for CO_2_ hydrogenation to methanol for the CP catalyst is ca. 20% lower than that previously estimated for pure In_2_O_3_^22^, whereas that of the RWGS reaction is equivalent. The microkinetic model (Supplementary Table 6) indicates that the apparent activation energy for methanol production is lowered by 56 or 4 kJ mol^–1^ when 1 or 2 exposed Pd atoms are present, respectively, and the RWGS reaction is hindered by a barrier higher by at least + 80 kJ mol^–1^ compared with In_2_O_3_. This means that competing transformation mainly occurs on unpromoted In_3_O_5_^22^ and is effectively blocked by the existence of 1 or 2 exposed Pd atoms at promoted sites. When 3 or more exposed Pd atoms are present, the active site starts behaving like metallic palladium, as the barrier of the RWGS reaction is decreased by –7 kJ mol^–1^ and methanol production is hindered. For the DI sample, methanol synthesis was facilitated to a comparable extent, but the RWGS reaction was also favored (activation energy decreased by ca. 10%, Fig. 5a), supporting the dual beneficial-detrimental action of palladium nanoparticles. In line with these results, a low activation energy for the parasitic reaction was also determined for Pd/TiO_2_. Similarly, In_2_O_3_ surfaces containing three or more exposed Pd atoms form CO rather than methanol and the Pd(111) surface is associated with the lowest activation barrier for the RWGS reaction of all models (Supplementary Table 6). The experimental measurements additionally indicate that a fourfold higher methanol *STY* (1.01 g_MeOH_ h^−1^ g_cat_^−1^, CO_2_ conversion = 9.7%, methanol selectivity = 78%) can be attained over the CP material at a 20-K lower temperature than for In_2_O_3_, implying higher productivity with lower energy input. The CP and equilibrated DI catalysts were further tested at variable partial pressures of reactants and products to derive their reaction orders (Table 1, Supplementary Figs. 22 and 23). The values obtained for CO_2_ and H_2_ for both catalysts are close to 0, thus being equal and substantially lower than for In_2_O_3_^22^, respectively. The reaction order for methanol was barely altered by palladium addition through either synthesis method, whereas the lower reaction order for H_2_ indicates superior H_2_ splitting ability over both systems. The negative rate dependence on the water pressure was greatly alleviated for the CP catalyst, whereas no significant change compared to In_2_O_3_ was determined for the DI sample. Facilitated water desorption represents a strong contributor to catalyst stability, as water-induced sintering is a major deactivation mechanism for the pure oxide^22^, whereby we speculate that surface OH groups condensation likely drives the coalescence of In_2_O_3_ crystals. This was corroborated by an extended test over the CP material aged for 200 h (Fig. 1b). Owing to the portion of palladium in the lattice, the material did not exhibit pronounced performance deterioration over 80 h (Fig. 1b). As expected, the methanol *STY* was overall inferior (0.54 versus 0.61 g_MeOH_ h^−1^ g_cat_^−1^) to the CP catalyst aged for 1 h since the methanol selectivity was reduced by the presence of the palladium particles (64% versus 80%, Supplementary Fig. 19). As the methanol production rate was almost constant upon altering the H_2_:CO_2_ ratio, the CP catalyst could be practically operated with a sub-stoichiometric H_2_ feed, thus greatly lowering the size of the recycle stream needed in the process, which will substantially reduce investment and operating costs for reactor, heat exchangers, and compressors^46^. Applying the optimal conditions identified in the kinetic analysis (H_2_:CO_2_ = 4, *P* = 5 MPa, *T* = 553 K, and *WHSV* = 48,000 cm^3^_STP_ h^−1^ g_cat_^−1^), catalyst durability was assessed in a 500-h test (Fig. 6). The CO_2_ conversion slightly dropped (from 9.7 to 9.2%) and the methanol selectivity increased (from 75 to 78%) in the first 200 h, whereas remaining unaltered until the end of the run. The average particle size of In_2_O_3_ increased by only 2 nm compared with CP 96 h (17 and 15 nm, respectively). Owing to the addition of TiO_2_ as a diluent in this test, which could not be separated from the used catalyst, changes in porous properties and vacancy density could not be investigated. The methanol *STY* was 0.96 g_MeOH_ h^−1^ g_cat_^−1^, which is, to our knowledge, the highest sustained productivity reported for CO_2_-based methanol synthesis over a heterogeneous catalyst (Table 2). Specifically, our catalyst reaches a 15%-higher methanol *STY* than the best performer reported and at a ca. 60% shorter residence time. The latter enables a reduction in reactor size by 60%, which is a strong gain for a prospective industrial process. Moreover, CP is a synthesis method that has been successfully implemented for catalyst manufacture at the large scale for numerous applications, whereas the polymer-assisted route used for the benchmark appears of limited industrial amenability.

**Table 1:** Reaction orders of reactants and products

Catalyst^a^	*n*_CO2_ [−]	*n*_H2_ [−]	*n*_MeOH_ [−]	*n*_H2O_ [−]
In_2_O_3_	−0.1	0.5–0.8	−0.2	−0.8
CP	−0.1	0.2	−0.3	−0.5
DI	−0.1	0.3	−0.2	−0.9

^a^Apparent reaction orders (*n*_i_) extracted by data regression from catalytic tests at variable partial pressures of CO_2_, H_2_, water, and methanol (Supplementary Figs. 22 and 23).

**Table 2:** Comparison of heterogeneous catalysts for CO_2_ hydrogenation to methanol

Catalyst	*T* [K]	*P* [MPa]	*WHSV* (cm^3^ h^−1^ g_cat_^−1^)	Initial *S*_MeOH_ (%)	Initial *STY*_MeOH_ (g_MeOH_ h^−1^ g_cat_^−1^)	Reference
Pd–In_2_O_3_ CP	553	5	48,000	78	1.01^a^	This work
Pd–In_2_O_3_ CP	553	5	24,000	75	0.61	This work
ZnO−ZrO_2_	593	5	24,000	86	0.71	^10^
Pd−Zn	543	2	18,000	70	0.60	^56^
LDH30−Ga	543	4.5	18,000	49	0.59	^57^
PdP−In_2_O_3_	593	5	21,000	67	0.89	^28^
CeO_2_ MoPK/SiO_2_	503	3.1	10,800	76	0.41	^58^
PdIn−In_2_O_3_/SiO_2_	573	5.0	63’000	24	ca. 0.3	^44^

^a^Sustained at 0.96 g_MeOH_ h^−1^ g_cat_^−1^ after 500 h on stream

## Discussion

We successfully produced a palladium-promoted In_2_O_3_ catalyst featuring palladium atomically dispersed in the oxide matrix by CP and a reference system comprising palladium nanoparticles by DI, the first showing a stable activity enhancement compared with bulk In_2_O_3_ exclusively directed to methanol production and the second displaying a decaying improvement of both methanol and CO formation. Extensive characterization by a battery of techniques, including solid-state ^115^In NMR, here introduced as a novel technique to elucidate vacancy formation and short-range crystallinity in (palladium-promoted) In_2_O_3_ catalysts, and theoretical modeling provided sound understanding of the structure and functioning of the active sites in the two systems. In the CP catalyst, embedding one Pd atom in the In_2_O_3_ matrix enables a controlled growth of extra-lattice atoms, leading to the stabilization of low-nuclearity palladium clusters. The number of vacancies does not increase but their electronic properties are strongly modified, permitting improved H_2_ dissociation. The small size of the palladium cluster is crucial to curtail the RWGS reaction on palladium sites, which is relevant already for clusters of 3 extra-lattice promoter atoms. In the DI catalyst, palladium steadily agglomerates with time on stream forming particles. These aid methanol production by supplying activated hydrogen that fosters the formation of surface vacancies on In_2_O_3_ and the hydrogenation of the CO_2_ therein adsorbed. Still, they consume a significant part of the H_2_ split by converting CO_2_ into CO on their own surface (Fig. 5b). These contrasting palladium speciations also explain the profoundly diverse time-on-stream behaviors of the two catalysts. Palladium embedded into indium oxide attracts the scarce palladium in pockets, avoiding its clustering, and facilitates water desorption minimizing In_2_O_3_ sintering, whereas palladium deposited onto In_2_O_3_ easily agglomerates and leads to catalyst sintering by spilling too much activated H_2_ to the oxide, causing over-reduction, and by excessively retaining water, provoking crystals coalescence. Hence, anchoring of palladium clusters to the In_2_O_3_ lattice is an unprecedented approach to effectively enhance activity and selectivity, and more importantly, to maintain the promotional effect in the long term, an aspect fully disregarded in previous studies on palladium-promoted In_2_O_3_ systems. In addition, low-nuclearity palladium clusters grant the possibility to operate the catalyst at reduced temperature, with hydrogen-lean feeds, and in the presence of greater amounts of water, altogether implying strong economic and ecologic benefits at a process level. Overall, our strategy to engineer promotion at the atomic scale marks a step ahead toward green methanol production and holds great general potential for tailoring new or existing promoted systems in current and emerging applications of heterogeneous catalysis.

## Methods

### Catalyst preparation

To obtain palladium-promoted (0.25–10 wt.% Pd) In_2_O_3_ catalysts by co-precipitation, In(NO_3_)_3_·*x*H_2_O (3.51, 3.50, 3.48, 3.47, 3.46, 3.45, 3.40, or 3.20 g, Sigma-Aldrich, 99.99%, *x* = 6.9) and Pd(NO_3_)_2_·*x*H_2_O (4.63, 11.6, 23.15, 34.8, 46.30 69.6, 162.5, or 463 mg, Sigma-Aldrich, > 99.99% metals basis, *x* = 5.5) were dissolved in deionized water (50 cm^3^) in a round-bottomed flask (250 cm^3^). Na_2_CO_3_ (10.0 g) was dissolved in deionized water (100 cm^3^) and added dropwise (ca. 25 cm^3^, 3 cm^3^ min^−1^) to the metals solution under stirring at room temperature to reach a pH value of 9.2. After aging the resulting slurry for 1 h, deionized water was added (50 cm^3^). Then, the precipitate was recovered by high-pressure filtration, washed with deionized water (three times, 500 cm^3^ each time), and dried in a vacuum oven (1.5 kPa, 323 K, 1.5 h). To produce palladium-containing (0.25–3.5 wt.% Pd) In_2_O_3_ catalysts by dry impregnation, a round-bottomed flask (25 cm^3^) was loaded with Pd(NO_3_)_2_·*x*H_2_O (7.8, 23.5, or 109.4 mg) and deionized water (0.39 g). After adding In_2_O_3_ (1.00 g) and five stainless-steel spheres (radius = 3 mm), the flask was rotated (ca. 45 r.p.m.) using a Büchi R-114 rotary evaporator at room temperature and pressure. After 12 h, the pressure was lowered to 2 kPa and the temperature raised to 333 K for 1 h to allow the evaporation of the solvent. Catalysts were calcined for 3 h at 573 K (2 K min^−1^) in static air prior to characterization or catalytic testing. The syntheses of all other materials reported in this study are detailed in the Supplementary Methods.

### Catalyst characterization

The metal content of the catalysts was determined by inductively coupled plasma optical emission spectrometry. When Na_2_CO_3_ was used as the precipitating agent, the absence of sodium in calcined materials was confirmed by X-ray fluorescence spectroscopy as well as by STEM-EDX and XPS for selected materials. The water amount in In and Pd precursors was derived from thermogravimetric analysis. Porous and structural properties of the catalysts were assessed by N_2_ sorption and powder XRD. Palladium dispersion, speciation and coordination geometry, and reducibility were accessed by volumetric chemisorption of H_2_ and CO, CO-DRIFTS, STEM-EDX, and HRTEM, X-ray absorption spectroscopy (XAS), TOF-SIMS, and by TPR. Surface concentration and oxidation states of C, O, Pd, and In were determined by XPS. Oxygen vacancies and CO_2_ adsorption capacity were characterized by XPS, ^115^In NMR, and TPR and TPD methods. Details to the characterization techniques are provided in the Supplementary Methods and in Supplementary Fig. 24.

### Catalytic testing

CO_2_ hydrogenation to methanol was conducted in a high-pressure continuous-flow fixed-bed reactor setup comprising mass flow controllers to feed gases (Bronkhorst, El-Flow F-201CV), pneumatic valves, a reactor with an inner diameter of 2.2 mm housed in an electrically-heated aluminum brass furnace, a pressure transducer, a burst plate calibrated at 6 MPa, a syringe pump to feed liquids (Chemyx Nexus 6000), an online gas chromatograph (Agilent 7890 A equipped with Agilent DB-1 and GS-GasPro columns), and a computer control by a custom protocol within the LabView software. In a typical test, the reactor was loaded with 100 mg of catalyst with a particle size of 100–125 μm, which was held in place by a bed of quartz wool and heated from ambient temperature to 553 K (5 K min^−1^) at 5 MPa under a He flow (20 cm^3^ min^−1^). After 3 h, the gas flow was switched to the reactant mixture (40 cm^3^ min^−1^) comprising H_2_ and CO_2_ (Messer, 99.997% and 99.999%, respectively) in a molar ratio of 4:1. For kinetic tests, temperature (473–653 K) and inlet partial pressures of reagents (*p*_H2_ = 3.5–4.5 MPa, *p*_CO2_ = 0.5–1.5 MPa) and products (*p*_MeOH_ and *p*_H2O_ = 0.05–0.25 MPa) were varied at a doubled *WHSV* attained by loading only 50 mg of catalyst diluted in 50 mg of TiO_2_ (100–125 μm, Sigma-Aldrich, > 99.9%). Water (ABCR-Chemicals, HPLC grade) or methanol (Sigma-Aldrich, > 99.9%, anhydrous) were fed to the reactor inlet by a high-pressure syringe pump (Chemyx, Nexus 6000). The effluent stream was sampled every 12 min and analyzed by online gas chromatography. Data evaluation procedures are presented in the Supplementary Methods and details to all catalytic tests are compiled in Supplementary Table 7.

### DFT

DFT simulations were performed using the Vienna Ab initio Simulation Package (VASP)^47,48^ using the Perdew–Burke–Ernzerhof density functional^49^. Valence electrons were described with a plane-wave energy cutoff of 500 eV, whereas core electrons were represented by projector augmented-wave pseudopotentials^50^. The In_2_O_3_(111) surface was modeled as a p(1 × 1) slab containing five O−In−O trilayers and optimized using a Γ-centered (3 × 3 × 1) *k*-point mesh. In each surface, the three outermost O−In−O trilayers were allowed to relax, whereas the two bottommost layers were fixed in their bulk positions. The initial structure of the CP catalyst was modeled by substituting an In atom in the In_3_O_5_(O) ensemble by a Pd atom. Three further structures include the addition of 1–3 Pd atoms bonded to the Pd atom in the oxide lattice. For the DI catalyst, 1–3 Pd atoms were added in the concave zone of the In_2_O_3_(111) surface. For both types of catalysts, O vacancy formation and palladium migration were explored. Energy profiles for CO_2_ hydrogenation to methanol and CO were calculated using the most feasible paths identified in our previous study^22^ on all CP and DI models, including the water-assisted step in the RWGS. To reduce the number of steps, the adsorption of the third H_2_ molecule was moved after H_2_COOH formation. The reaction network occurring on the Pd(111) surface, representing the palladium nanoparticles in the equilibrated DI sample, was also taken from previous studies^35,45^. The Gibbs free energies of the adsorbed species considered only vibrational contributions. For gas phase, translational and rotational terms were also included and obtained from Gaussian 09^51^. The reaction network was interrogated through a microkinetic model based on a differential reactor under steady-state conditions. The kinetic parameters were modeled from transition state theory, whereas adsorption processes followed the Hertz–Knudsen equation^46^. The resulting system of equations was resolved in Maple using a floating-point precision of 96 digits. The temperatures and pressures of operation were chosen to match the experimental ones. For relevant systems, the XPS core-level Pd3*d* shifts were computed in VASP, using metallic Pd as reference^52^, and were compared with the Bader charges (Supplementary Table 9). EXAFS spectra were obtained from XAS simulations attained using feff8^53^ after processing with Athena^54^ in the same way as for the experimental data and subsequent normalization.

## Supplementary information

Supplementary Information

Peer Review

## Data Availability

The authors declare that the data supporting the findings of this study are available within the article and its Supplementary Information file. All DFT data and the corresponding structures are accessible at the ioChem-BD database^55^ following the link: 10.19061/iochem-bd-1-106. All other relevant source data are available from the corresponding author upon reasonable request.
