# Supplementary material for: Atomic-scale engineering of indium oxide promotion by palladium for methanol production via CO2 hydrogenation
Source: Nat Commun. 2019 Jul 29;10:3377. doi: 10.1038/s41467-019-11349-9 (PMC6662860; doi:10.1038/s41467-019-11349-9)
Supplement: Supplementary file 1 — Supplementary Information [file 41467_2019_11349_MOESM1_ESM.pdf]

Supplementary Information

Atomic-scale engineering of indium oxide promotion by palladium for methanol production *via* CO_2 hydrogenation

Frei *et al*.

Table of contents

Supplementary Methods 2

Supplementary Figures 8

Supplementary Tables 28

Supplementary References 36

Supplementary Methods

Additional details on catalyst preparation

In_2O_3 was prepared through the controlled calcination of precipitated (P) $In(OH)_3$, based on a protocol reported earlier.[1] Briefly, $In(NO_3)_3 \cdot xH_2O$ (15.5 g, Sigma-Aldrich, 99.99%, $x = 6.9$) and Na_2CO_3 (20.0 g, Merck, >99%) were separately dissolved in deionized water (235 and 200 cm^3, respectively). The sodium carbonate solution was added to the indium-containing solution under magnetic stirring at ambient temperature until a pH value of 9.2 was reached (*ca.* 158 cm^3, 9 cm^3 min^{-1}). After aging for 60 min, the precipitate was recovered by high-pressure filtration, washed with deionized water (3 times, 1 L each time), and dried in a vacuum oven (2 kPa, 323 K, 12 h). The thus obtained hydroxide was calcined in static air for 3 h at 573 K (heating rate = 2 K min^{-1}) to yield nanocrystalline In_2O_3.

In addition to the syntheses described in the main manuscript, palladium was also deposited onto In_2O_3 by wet impregnation (WI), a sol-gel method (SG), and spray deposition (SD). In the first case, $Pd(NO_3)_2 \cdot xH_2O$ (23.5 mg, Sigma-Aldrich, >99.99% metals basis, $x = 5.5$) was dissolved in deionized water (50 cm^3) and In_2O_3 (1.00 g) was added to the mixture, which was then stirred at ambient temperature for 1 h. Following the evaporation of the solvent in a Büchi R-114 rotary evaporator, the sample was dried in a vacuum oven (1.5 kPa, 323 K, 1.5 h) and calcined in static air for 3 h at 373 K (2 K min^{-1}). For SG, $In(NO_3)_3 \cdot xH_2O$ (3.48 g) and $Pd(NO_3)_2 \cdot xH_2O$ (34.8 mg) were loaded with nitric acid (0.19 g, Fisher Scientific UK, 65 wt.% in H_2O) and deionized water (1.82 g) in a round-bottomed flask (250 cm^3) and stirred until complete dissolution was observed by eye. A 66.7 wt.% citric acid solution was prepared by dissolving citric acid (1.00 g, Sigma-Aldrich, >99.5%) in deionized water (0.5 cm^3) and added to the solution of metal nitrates. Water was evaporated at 333 K (10 K min^{-1}) under magnetic stirring during 3 h, yielding a highly viscous gel, which was dried in a vacuum oven (1.5 kPa, 323 K, 1.5 h), followed by calcination in a tubular oven, flowing dried air (0.12 m^3 h^{-1}) for 3 h at 773 K (2 K min^{-1}). SD was carried out in a Büchi Mini Spray Dryer B-290. After priming the spray dryer with deionized water, a slurry containing In_2O_3 (1 g, particle size <50 μm), deionized water (10 cm^3), and a $Pd(NO_3)_2$ solution (88.2 mg, 8.5 wt.% Pd in diluted nitric acid, ABCR-Chemicals) was fed to the dryer. The following parameters were set on the instrument: aspiration = 80% (*ca.* 28 m^3 h^{-1}), spray gas (air) flow = 0.6 m^3 h^{-1} at 0.6 MPa, pump = 10% (*ca.* 1 cm^3 min^{-1}), inlet temperature = 593 K, and nozzle cleaner = 0. The sample was unloaded from the collector and calcined in static air for 3 h at 573 K (2 K min^{-1}).

Pd-In alloys, evaluated as a catalyst and serving as a reference for X-ray absorption spectroscopy (XAS) studies, were attained by a chemical reduction (CR) method. $Pd(NO_3)_2 \cdot xH_2O$ (53.7 or 5.37 mg) and $In(NO_3)_3 \cdot xH_2O$ (57.4 or 109 mg) were placed in a Büchi glass autoclave with dodecane (150 cm^3, Acros, 99%) and vigorously stirred (*ca.* 1000 rpm). Air was flushed out from the reactor by flowing N_2 (*ca.* 200 cm^3 min^{-1}) for 20 min, thereafter the autoclave was pressurized to 0.15 MPa and heated to 455 K in the same flow of nitrogen. After 2.5 h, the pressure was increased to 0.5 MPa and the gas feed was changed to 5 mol% H_2 in N_2 (Messer, 99.999% each, 200 cm^3 min^{-1}) for 16 h. The formed solid was separated from the solvent by high-pressure filtration and dried in a vacuum oven (1.5 kPa, 323 K, 24 h).

Pd-TiO_2 catalysts were obtained by DI, following the method reported in the main manuscript, using TiO_2 (Sigma-Aldrich, >99.8% metal bases) instead of In_2O_3, and by CP. In the latter case, the $In(NO_3)_3$ precursor was replaced by titanium isopropoxide (5.6 cm^3, ABCR-Chemicals, 98%) and anhydrous ethanol was used as the solvent to prevent hydrolysis of the titanium precursor.

Ir-, Ag-, Au-, Ru-, Cu-, and Pt-In_2O_3 were produced by the CP method outlined in the main manuscript. Here, $IrCl_3$ (17.4 mg, ABCR-Chemicals, 99.9%), $AgNO_3$ (17.7 mg, ABCR, 99.9%), $HAuCl_4$ (19.4 mg, ABCR, 99.9%), $RuCl_3 \cdot xH_2O$ (30.1 mg, Sigma-Aldrich, 99.98%, $x = 3.5$), $Cu(NO_3)_3 \cdot xH_2O$ (42.8 mg, 99.9%, Merck, $x = 3$), and PtK_2Cl_4 (23.9 mg, ABCR, 99.9%) were used as the metal precursors, respectively.

PdO, serving as another reference for XAS measurements, was prepared by the controlled decomposition of $Pd(NO_3)_2 \cdot xH_2O$ upon heating in static air for 5 h at 1073 K (10 K min^{-1}).

Characterization

Inductively coupled plasma-optical emission spectrometry (**ICP-OES**) was conducted using a Horiba Ultra 2 instrument equipped with photomultiplier tube detector to assess the actual metal content of all promoted materials. Prior to analysis, the specimens were dissolved in *aqua regia* and the resulting solutions were diluted with twice-distilled water (ABCR-Chemicals, HPLC grade). X-ray fluorescence spectroscopy (**XRF**) was performed using an Orbis Micro-EDXRF spectrometer equipped with a Rh source operated at 35 kV and 500 μA and a silicon drift detector. The water content of the commercial metal precursors used was determined by thermogravimetric analysis (**TGA**) using a Linseis TGA PT1600 thermobalance. The solids (*ca.* 25 mg) were placed in an alumina crucible and heated from

ambient temperature to 1073 K (5 K min^{-1}) in flowing Ar (200 cm^3 min^{-1}). The water content was associated to the weight loss between 293-393 K. **Nitrogen sorption** at 77 K was conducted using a Micromeritics TriStar II analyzer. Prior to the measurements, the samples were degassed at 573 K under vacuum for 3 h. The total surface area (S_{BET}) was determined using the Brunauer-Emmet-Teller (BET) model. Powder X-ray diffraction (**XRD**) was performed in a PANalytical X'Pert PRO-MPD diffractometer operated in Bragg-Brentano geometry using Ni-filtered Cu Kα (λ = 0.1541 nm) radiation. Data were recorded in the range of 10-70° 2θ with an angular step size of 0.025° and a counting time of 12 s per step. Temperature-programmed desorption of CO and CO_2 (**CO-TPD**, **CO_2-TPD**) and reduction with CO and H_2 (**CO-TPR**, **H_2-TPR**) were carried out in a Micromeritics AutoChem HP II analyzer coupled to a Pfeiffer OmniStar mass spectrometer (monitoring m/z = 2, 18, 28, 44). The conditions applied to these analyses are detailed in **Supplementary Table 8**. The mass spectrometry signal for CO was corrected from the contribution of CO produced by fragmentation of CO_2. **Volumetric chemisorption** of H_2 and CO was performed at 293 K using a Quantachrome Autosorb-1C gas sorption system. Prior to the measurements, samples were dried at 373 K in flowing nitrogen for 2 h, followed by reduction in H_2 (50 cm^3 min^{-1}) at 553 K for 2 h (ramp rate = 5 K min^{-1}), evacuation at 553 K for 2 h, and cooling down to the analysis temperature in vacuum. To correct for weak interactions, the dual-isotherm method was applied. In this approach, the adsorption isotherm is recorded twice: the first isotherm includes strong and weak interactions and the second isotherm, recorded after evacuation of the catalyst at analysis temperature, only comprises weakly adsorbed probe molecules. The difference between these two isotherms provides the amount of gas strongly adsorbed that is representative of the monolayer capacity.

Stoichiometries of Pd:H_2= 2:1 and of Pd:CO = 1:1 were used in the calculations of the dispersion. Diffuse reflectance Fourier transform infrared spectroscopy of adsorbed CO (**CO-DRIFTS**) was conducted in a Bruker Equinox 55 spectrometer equipped with a Harrick Praying Mantis cell in the range of 650-4000 cm^{-1}, with 2 cm^{-1} optical resolution, and accumulation of 300 scans. The sample was diluted 5 times by weight with silicon powder (ABCR-Chemicals, 99.995%) and placed in the cell, followed by heating to 553 K (10 K min^{-1}, 30 min) in a flow (10 cm^3 min^{-1}) of H_2 (5 mol% in He). Thereafter the flow was changed to Ar (10 cm^3 min^{-1}) and the sample cooled to room temperature. CO adsorption was conducted subsequently in a flow (10 cm^3 min^{-1}) of CO (5 mol% in Ar) for 30 min with subsequent Ar purging (10 cm^3 min^{-1}) for 30 min. Spectra were recorded before and after the chemisorption

step, and the first subtracted from the latter. Time of flight secondary ion mass spectroscopy (**TOF-SIMS**) was measured in a IONTOF TOF.SIMS[5] instrument in spectroscopy mode using Bi_3^+ ions accelerated to 25 keV. Two different areas of 84×84 μm^2 were analyzed and the reported data comprises the average of the two scans. Scanning transmission electron microscopy imaging and energy dispersive X-ray spectroscopy (**STEM-EDX**) as well as high resolution transmission electron microscopy (**HRTEM**) were performed using a Talos F200X instrument operated at 200 kV and equipped with a FEI SuperX detector. X-ray photoelectron spectroscopy (**XPS**) was conducted in a Physical Electronics Instruments Quantum 2000 spectrometer using monochromatic Al Kα radiation generated from an electron beam operated at 15 kV and 32.3 W. The spectra were collected under ultra-high vacuum conditions (residual pressure = $5\cdot10^{-8}$ Pa) at a pass energy of 46.95 eV. All spectra were referenced to the C1*s* peak at 284.8 eV. Although catalysts were extracted from the reactor in inert atmosphere, the design of the instrument made a brief (<2 min) exposure to air upon sample introduction unavoidable. Selected catalysts were sputtered with Ar^+ ions to remove *ca*. 4 nm of their surface to access information about their bulk. Pd K-edge X-ray absorption spectroscopy (**XAS**) was measured at the SuperXAS beamline at the Swiss Light Source (Villigen, Switzerland).[2] The incident beam was provided by the 2.9-T super-bent source and was collimated by a Pt-coated mirror at 2.5 mrad and focused by a toroidal Pt-coated mirror. The energy was selected by a Si(111) channel-cut monochromator, and calibrated using a Pd foil (24.350 keV), which was measured simultaneously with the specimen of interest. Samples were transferred from the reactor to a quartz capillary under inert atmosphere. The incident X-ray beam was focused on a 0.1×0.1 mm^2 spot. XAS data was acquired in fluorescence mode using a 5-element silicon drift detector. The spectra were calibrated and normalized to the edge jump of 1 using the Demeter software package.[3] k^3-weighted extended X-ray absorption fine structure (**EXAFS**) spectra were fitted in the *k*- and *R*-windows indicated in **Supplementary Figure 4**. An amplitude reduction factor (S_0^2) of 0.82 was determined by fitting of the EXAFS spectrum of a Pd foil. The scattering paths for the fitting were produced using known crystallographic structures of metallic Pd, tetragonal PdO, and cubic In_2O_3. Solid state nuclear magnetic resonance spectroscopy of the ^{115}In nucleus (**^{115}In NMR**) was conducted using a Bruker Avance III HD 700 MHz spectrometer equipped with a 16.4 T standard bore magnet operated at 152.5-154.5 MHz. Spectra were collected using 16,384 accumulations with a pulse length of 8 ms and a recycle delay of 1 s. Due to the high spectral width caused by the quadrupolar character of In nuclei and the technical limitation of generating a 2 MHz pulse, it is not possible

to excite the whole spectrum of ^{115}In with a single centered pulse. In order to overcome this limitation, the measurement was repeated at different frequencies in the 152.5-154.5 MHz range (280-kHz steps). The collected sub-spectra were combined together in MATLAB using the matNMR tool[4] (**Supplementary Figure 24**), with a custom-programmed script to Fourier transform free induction decay (FID) signal, followed by combination of the sub-spectra considering the non-uniformity of the excitation pulses. Since the lack in uniformity at the pulse-end causes the intensity of the ending signals to be artificially weakened, measurements were chosen to have *ca*. 75% overlap with adjacent ones to obtain composite spectra as accurate as possible. All spectra were normalized to their respective highest signal intensity.

Evaluation of the catalytic data

The response factors F_i for each compound i in the effluent stream, respective to the internal standard (CH_4), in the gas chromatography analysis were determined by the equation

$$F_i = \frac{A_i / \dot{n}_i^{\text{in}}}{A_{\text{CH}_4} / \dot{n}_{\text{CH}_4}^{\text{in}}} \tag{1}$$

where A_i is the integrated area determined for the peak of compound i in the gas chromatogram and $\dot{n}_i^{\text{in}}$ corresponds to its known molar flow rate at the reactor inlet. The response factors of the analytes i were calculated as the average of 5 points around the expected concentrations of the respective analyte. Under reaction, the unknown effluent molar flow rate $\dot{n}_i^{\text{out}}$ was determined using the equation

$$\dot{n}_i^{\text{out}} = \frac{A_i \times F_i}{A_{\text{CH}_4}} \times \dot{n}_{\text{CH}_4}^{\text{in}}, \ \text{mol}_i \ \text{h}^{-1} \tag{2}$$

Conversion (X_i), selectivity (S_i), and production rate (r_i) were calculated applying equations 3-5

$$X_i = \frac{\dot{n}_i^{\text{in}} - \dot{n}_i^{\text{out}}}{\dot{n}_i^{\text{in}}} \times 100, \ \% \tag{3}$$

$$S_i = \frac{\dot{n}_i^{\text{in}} - \dot{n}_i^{\text{out}}}{\dot{n}_{\text{CO}_2}^{\text{in}} - \dot{n}_{\text{CO}_2}^{\text{out}}} \times 100, \ \% \tag{4}$$

$$r_i = \frac{\dot{n}_i^{\text{in}} - \dot{n}_i^{\text{out}}}{w_{\text{cat}}}, \ \text{mol}_i \ \text{h}^{-1} \ \text{g}_{\text{cat}}^{-1} \tag{5}$$

where $\dot{n}_i$ corresponds to the molar flow rate of species i and w_{cat} is the catalyst weight. The methanol space-time yield (STY) is the product of r_{MeOH} and the molar weight of methanol (32.04 g mol^{-1}). Data reported correspond to the average of the 4 measurements preceding a specific time-on-stream, or to the average of 7 measurements collected during each individual condition when temperature or gas flows were altered. The carbon balance was determined for each experiment according to equation 6 and was found to be always within 3%.

$$\varepsilon_{\mathrm{C}} = \left(1 - \frac{\dot{n}_{\mathrm{CO_2}}^{\mathrm{out}} + \dot{n}_{\mathrm{MeOH}}^{\mathrm{out}} + \dot{n}_{\mathrm{CO}}^{\mathrm{out}}}{\dot{n}_{\mathrm{CO_2}}^{\mathrm{in}} + \dot{n}_{\mathrm{MeOH}}^{\mathrm{in}}}\right) \times 100,\ \% \qquad (6)$$

The absence of intra- and extraparticle diffusion limitations was corroborated by the fulfillment of the Weisz-Prater ($\Phi \sim 0.1$) and Carberry ($Ca \sim 0.01$) criteria at reaction conditions of T = 553 K, P = 5 MPa, molar H_2:CO_2 = 4, and $WHSV$ = 48,000 cm$^3_{\mathrm{STP}}$ h^{-1} g$_{\mathrm{cat}}^{-1}$.

Supplementary Figures

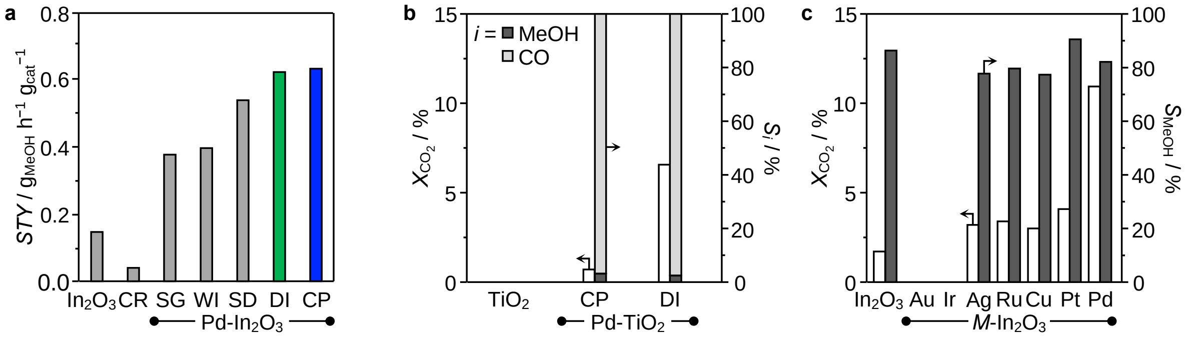

Supplementary Figure 1 | Catalytic performance in CO_2 hydrogenation of **a**, palladium-promoted In_2O_3 catalysts prepared by various synthetic approaches, **b**, Pd-TiO_2 catalysts obtained by DI or CP, and **c**, In_2O_3 catalysts promoted by various metals (*M*) attained by CP after 1 h on stream. The nominal metal loading in all the samples was 0.75 wt.%. Panel **c** shows that, although other typical hydrogenation metals (gold, iridium, silver, ruthenium, copper, and platinum) also exerted a promotional effect when incorporated into In_2O_3 by CP, none reached the level of palladium. Reaction conditions: $T = 553$ K, $P = 5$ MPa, $H_2{:}CO_2 = 4$, and $WHSV = 24{,}000$ cm^3_{STP} h^{-1} g_{cat}^{-1}.

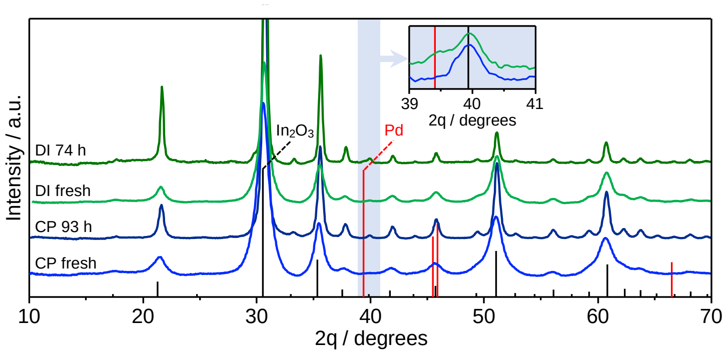

Supplementary Figure 2 | XRD patterns of the palladium-promoted In_2O_3 catalysts (0.75 wt.% Pd) obtained by CP and DI in fresh form and after use in CO_2 hydrogenation. Reference diffractograms of pure In_2O_3 and metallic palladium (ICDD 98-002-1994 and 98-001-4186, respectively) are shown with vertical lines. The inset depicts a magnified view of the region in which the most intense reflection of metallic palladium can be detected. Reaction conditions: $T = 553$ K, $P = 5$ MPa, $H_2{:}CO_2 = 4$, and $WHSV = 24{,}000$ cm^3_{STP} h^{-1} g_{cat}^{-1}.

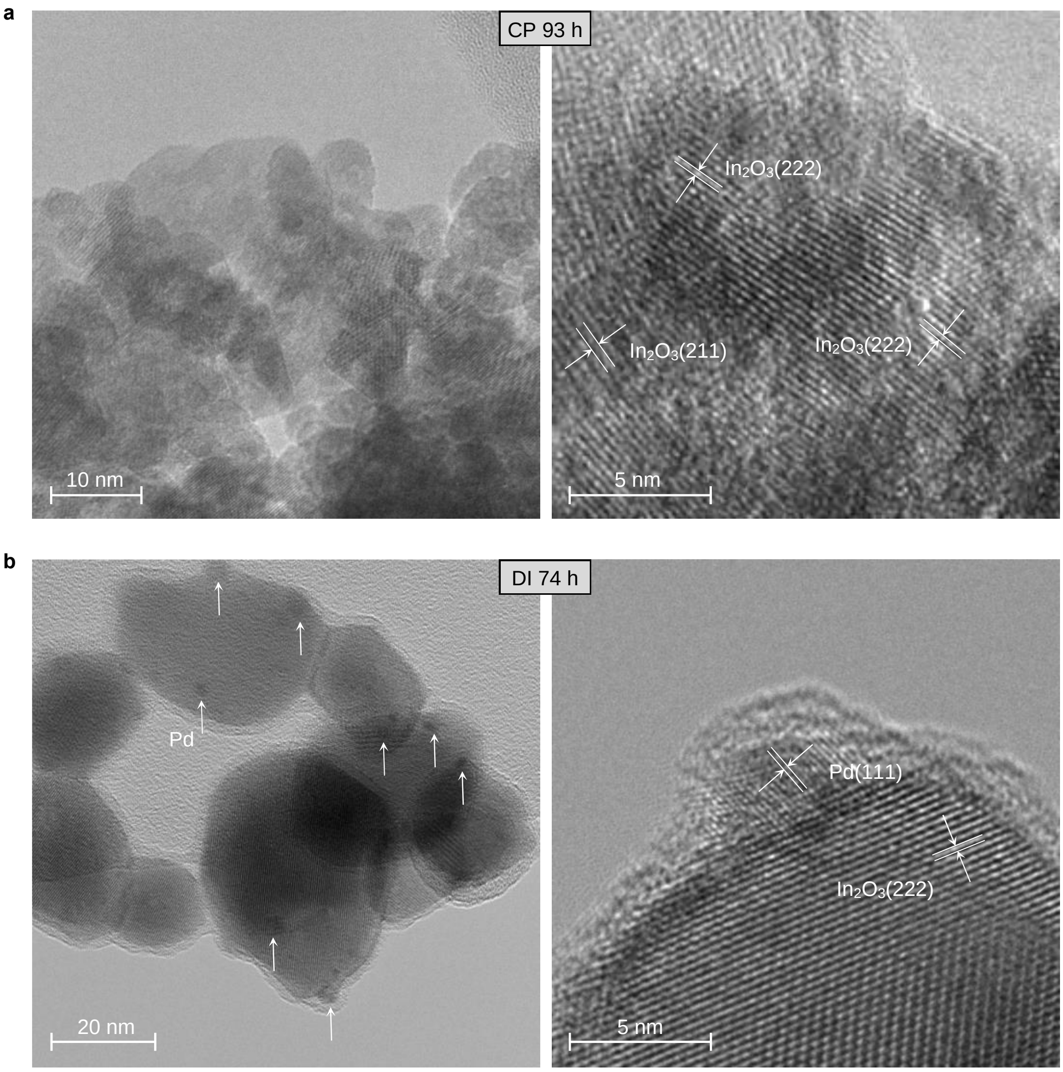

Supplementary Figure 3 | HRTEM images of the palladium-promoted In_2O_3 catalysts (0.75 wt.% Pd) prepared by **a**, CP and **b**, DI after use in CO_2 hydrogenation. Palladium nanoparticles were identified by contrast and fringe-analysis in the DI sample. Reaction conditions: T = 553 K, P = 5 MPa, H_2:CO_2 = 4, and $WHSV$ = 24,000 cm^3_{STP} h^{-1} g_{cat}^{-1}.

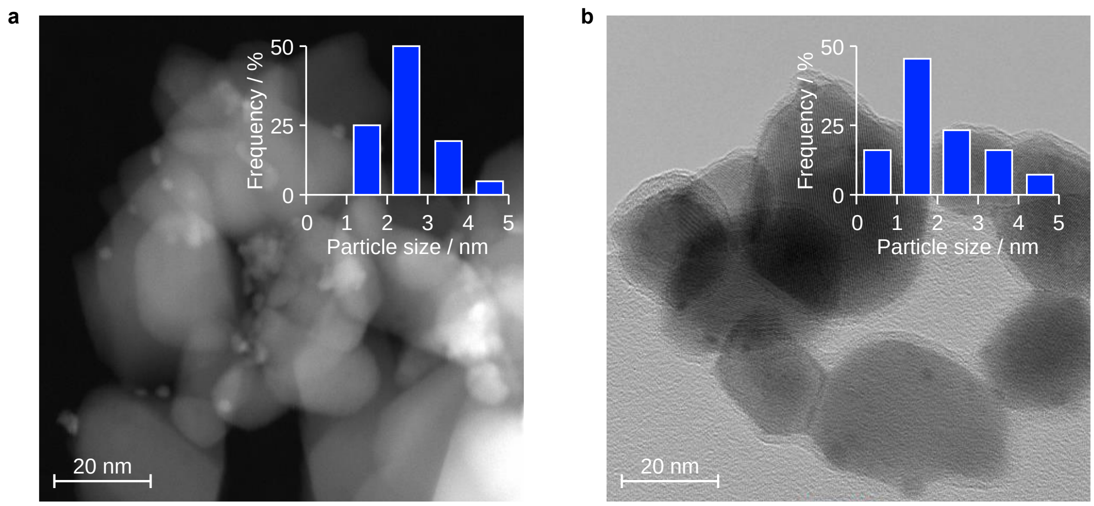

Supplementary Figure 4 | STEM image of the Pd/TiO_2 reference material. **b**, HRTEM image of the DI 74 h catalyst. The insets show the size distribution of palladium nanoparticles.

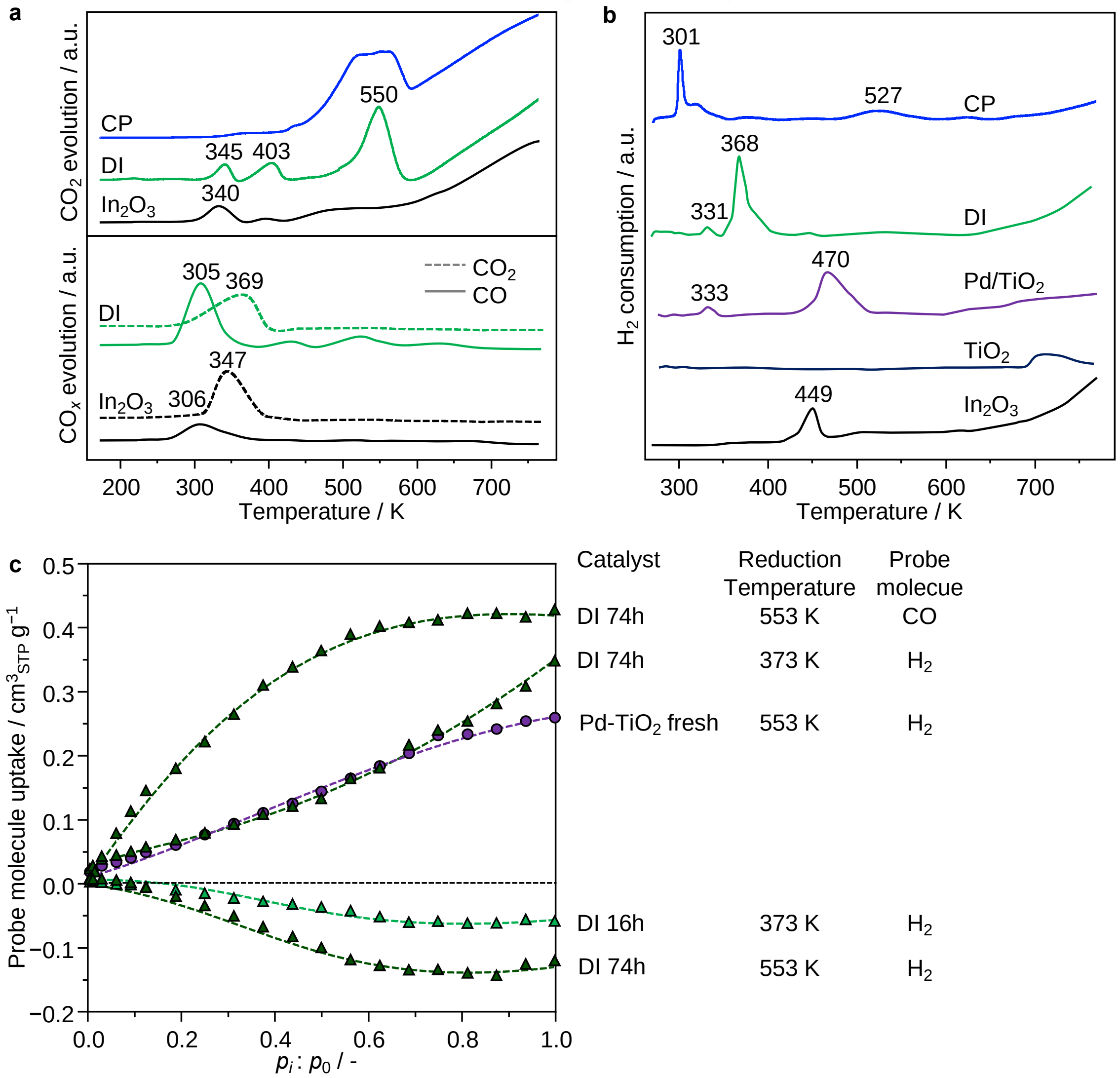

Supplementary Figure 5 | a CO-TPR of fresh CP and DI (top) and CO-TPD from DI (bottom) with In_2O_3 as a reference. **b**, H_2-TPR of CP, DI, In_2O_3 and Pd (0.75 wt.%)/TiO_2 prepared by DI. **c**, Isotherms obtained by volumetric chemisorption of H_2 and CO at 293 and 553 K for used DI catalysts and the reference Pd/TiO_2. CO-TPD of fresh DI indicates that CO chemisorption should be conducted at *ca.* 373 K to avoid interference from the oxide. Still, CO-TPR and CO-TPD show that CO already reacts with In_2O_3 at that temperature, which forces to perform the analysis at lower temperatures. Concerning H_2-TPR, the profiles evidence two main peaks between *ca.* 290-365 for CP and 323-393 K for DI and the start of a signal at *ca.* 643 K. Based on the H_2-TPR of In_2O_3, the sharp low-temperature signals are likely due to the reduction of the oxide surface, while the high-temperature H_2 consumption indicates that the bulk of the oxide starts to reduce. Although the H_2-TPR profile of Pd/TiO_2 shows peaks at 333 and 447 K related to the reduction of palladium nanoparticles and the oxide surface, respectively, some palladium could still contribute to the signal centered at 363 K for the DI catalyst. Accordingly,

pre-reduction and volumetric chemisorption with H_2 and CO were conducted at 373 and 293 K, respectively. Based on the negligible H_2 uptake by used samples, DI 74 h was analyzed again after a pretreatment at the reaction temperature to minimize the presence of adsorbates formed upon CO_2 hydrogenation, but the same outcome was obtained. CO chemisorption on the same sample after the high-temperature reduction led to a very low gas uptake too (palladium dispersion = 3.9%). We put forward strong metal support interactions (SMSI) and/or alloying of palladium with indium as plausible reasons for the dramatic change in electronic properties of palladium in our samples. H_2 chemisorption on Pd/TiO_2, which comprises palladium nanoparticles with an average size of *ca*. 3 nm (Figure R5), a catalyst for which the SMSI phenomenon has been widely documented,[5] also led to an estimated palladium dispersion of only 2%.

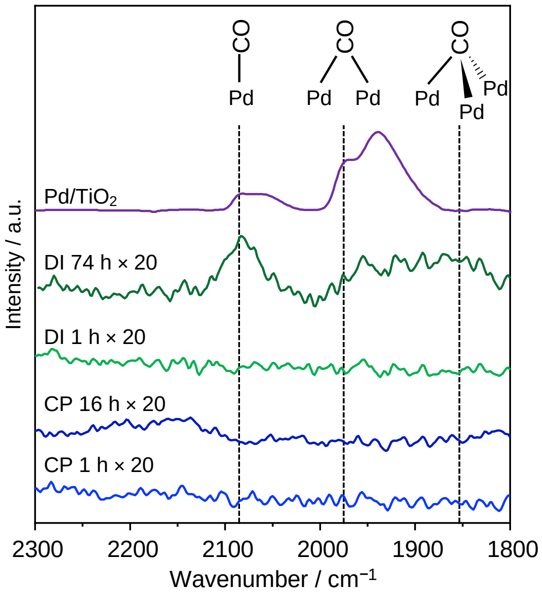

Supplementary Figure 6 | CO-DRIFT spectra acquired after CO adsorption on used CP and DI catalysts and the reference Pd/TiO_2 material. Expected locations of signals specific to linearly and two- and three-fold bound CO are indicated.

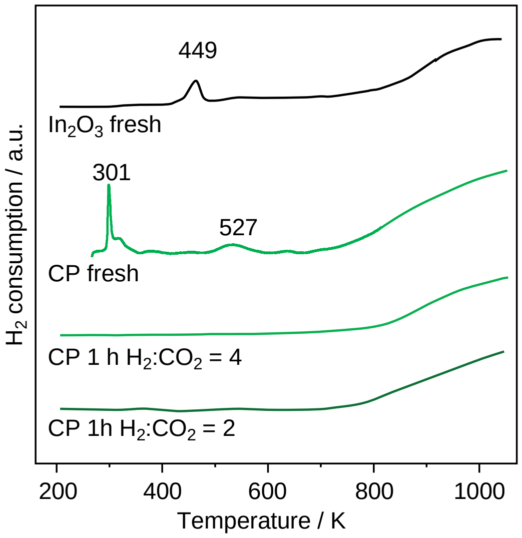

Supplementary Figure 7 | Profiles obtained upon H_2-TPR for the CP catalyst used for 1 h in CO_2 hydrogenation at distinct H_2:CO_2 ratios. The profiles for the CP catalyst and unpromoted In_2O_3 in fresh forms are shown as references. No reduction event associated with the surface of In_2O_3 or palladium was observed, which is expected below 800 K based on the profile obtained for the fresh CP sample and pure In_2O_3. This hints that the reducing power of hydrogen largely exceeds the oxidizing potential of CO_2 for the feed compositions explored.

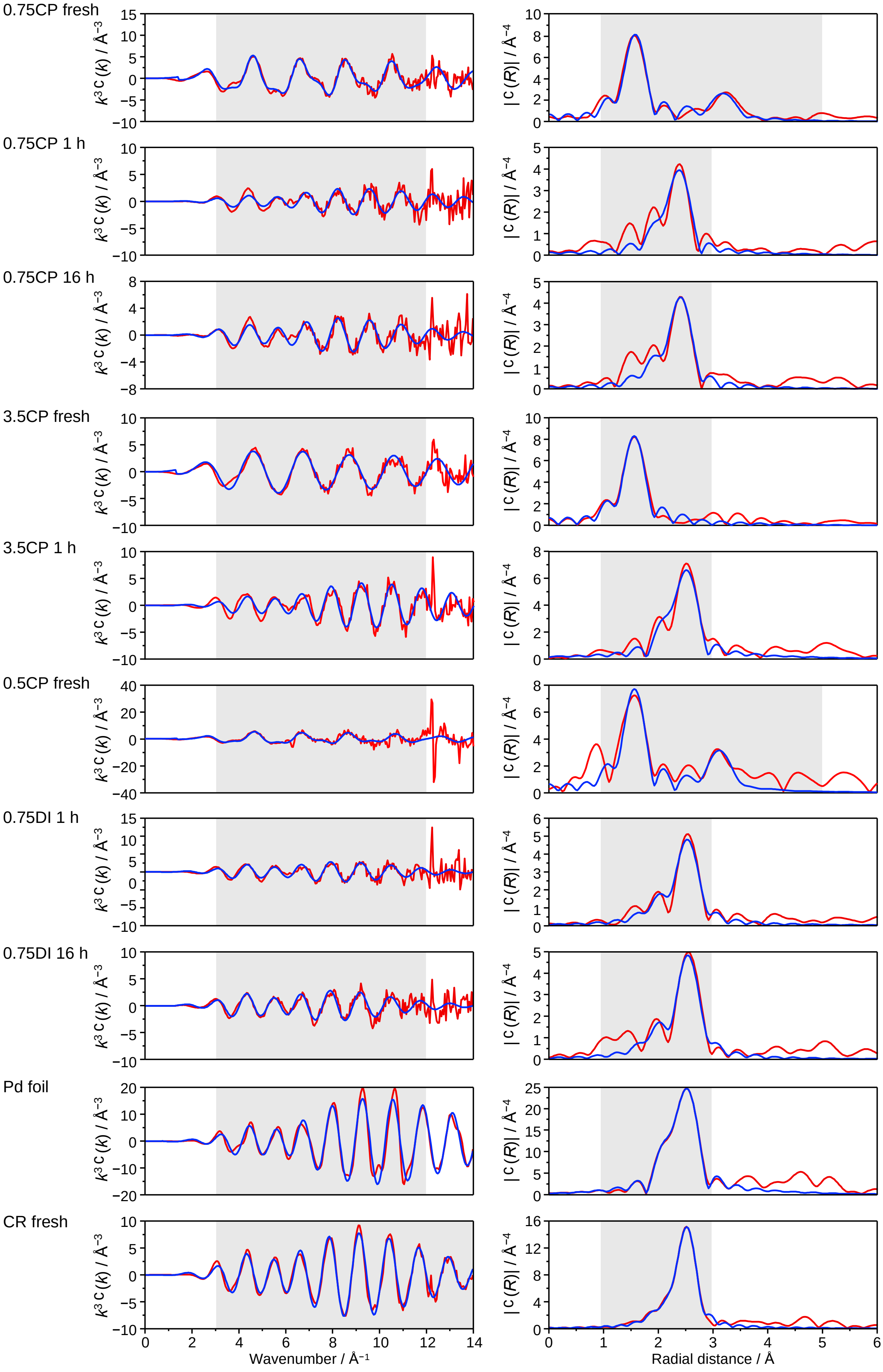
0.75CP fresh
0.75CP 1 h
0.75CP 16 h
3.5CP fresh
3.5CP 1 h
0.5CP fresh
0.75DI 1 h
0.75DI 16 h
Pd foil
CR fresh
$k^3\chi(k)$ / Å^{-3}
$|\chi(R)|$ / Å^{-4}
Wavenumber / Å^{-1}
Radial distance / Å

Supplementary Figure 8 | Model spectra (blue) relevant to the scattering paths specific to Pd-Pd, Pd-In, Pd-O, and Pd-O-In environments fitted to the acquired EXAFS spectra (red) for the palladium-promoted In_2O_3 catalysts obtained by CP and DI in fresh form and after use in CO_2 hydrogenation to extract the Pd coordination geometry. The number preceding the acronym of the synthesis in the catalysts' codes indicates the nominal palladium loading in wt.%. The spectra of palladium foil and the material prepared by CR are included as references. Data is presented both in the *k*-space (left) and *R*-space (right) and the region analyzed is marked in grey.

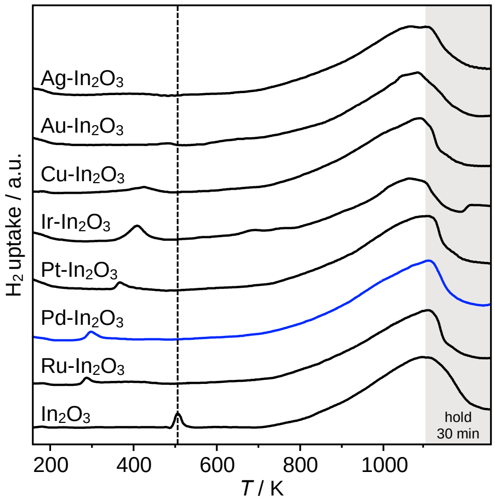

Supplementary Figure 9 | H_2-TPR profiles collected at 5 MPa of the catalysts shown in **Supplementary Figure 1c** in fresh form. In the presence of any promoter, surface reduction of In_2O_3 occurs at lower temperature compared to pure In_2O_3 (marked by the dashed line), but does not consume much more H_2. This suggests that all metals, when introduced by CP, facilitate the formation of vacancies, but do not substantially increase their amount.

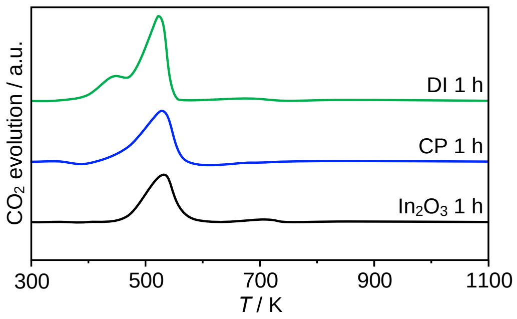

Supplementary Figure 10 | TPD of CO_2 from the palladium-promoted In_2O_3 catalysts (0.75 wt.% Pd) obtained by CP and DI and from pure In_2O_3 serving as a reference. The profiles were collected after quenching a 1-h reaction run by rapid cooling (−90 K min^{-1}). Reaction conditions: T = 553 K, P = 5 MPa, H_2:CO_2 = 4, and $WHSV$ = 24,000 cm^3_{STP} h^{-1} g_{cat}^{-1}.

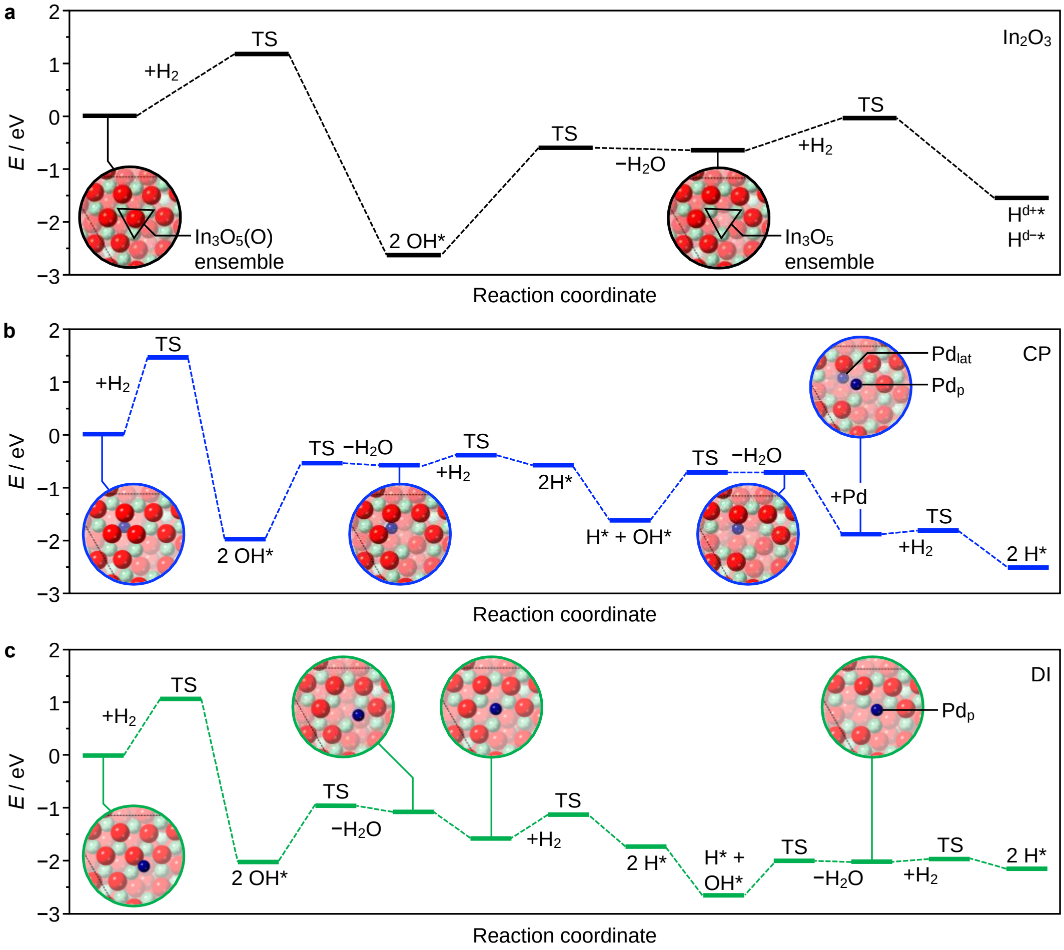

Supplementary Figure 11 | Energy profiles for the removal of the O atom in the center of the In_3O_5(O) ensemble and the subsequent activation of H_2 over surface models representing **a**, pure In_2O_3 and palladium-promoted In_2O_3 obtained by **b**, CP and **c**, DI. Intermediate surface restructuring, *i.e.*, migration of palladium atoms from the pocket indicated in **Figure 3g** (Pd_p)

and formation of a second O vacancy at the ensemble, was also considered for the CP and DI catalysts. The role of palladium in both promoted systems is two-fold: (i) the OH* species formed upon dissociative adsorption of H_2 is less stable than over In_2O_3, which favors water desorption and, hence, the generation of the vacancy within the ensemble, and (ii) H_2 activation for the reaction at the catalytic site becomes homolytic with virtually no energy barrier. It is worth noting that Pd_p atoms migrating to Pd atoms in the In_2O_3 lattice (Pd_{lat}) in the CP catalyst form a stable dimer (corresponding to model CP_b in **Figure 3g**). In contrast, Pd_p atoms moving to the active ensemble in the DI system (corresponding to model DI fresh in **Figure 3g**) remain highly mobile and are expected to cluster with neighboring Pd_p atoms in the course of the reaction (corresponding to model DI_a in **Figure 3g**).

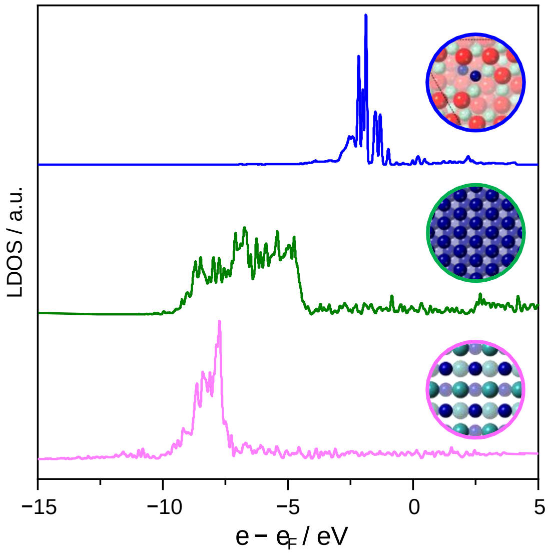

Supplementary Figure 12 | Local density of states (LDOS) of the exposed Pd atom in the CP_b material, a Pd atom on a Pd(111) surface, which represents the electronic structure of nanoparticles in the DI_c system, and a Pd atom in an InPd intermetallic as in ref. 6. O, Pd, and In atoms are colored in red, blue, and turquoise, respectively. Zero corresponds to the Fermi level in all cases.

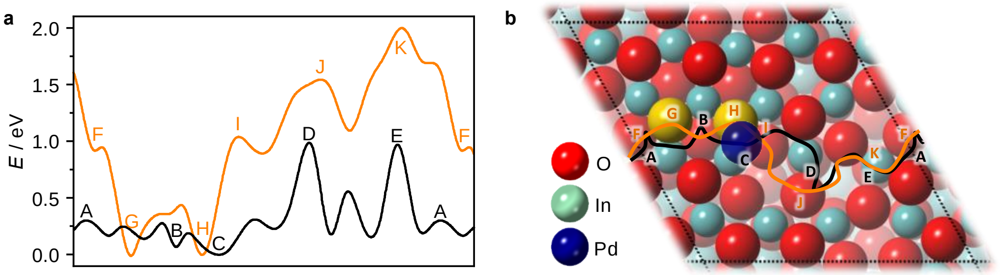

Supplementary Figure 13 | a, Potential energy profiles for Pd diffusion on an In_2O_3(111) surface without (black) and with two oxygen vacancies (orange, vacant sites generated when the yellow-highlighted O atoms are removed). **b**, Trajectory of the Pd atoms during the diffusion. The potential energy and positions of the most relevant states are indicated. All energies are referred to the respective global minima “C” (black) and “G” (orange).

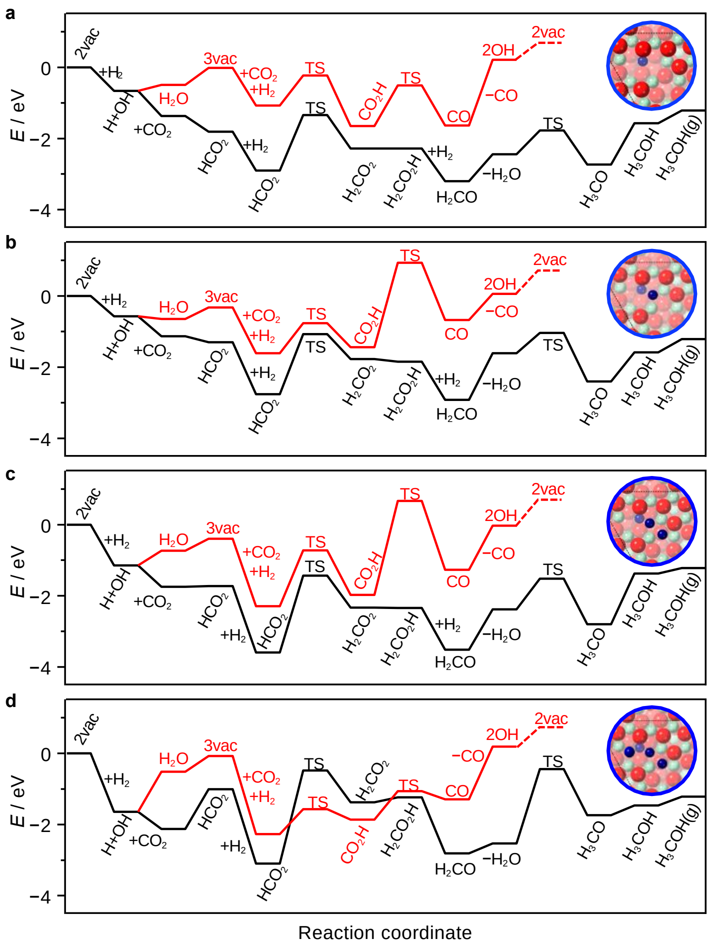

Supplementary Figure 14 | Energy profiles for CO_2 hydrogenation to methanol on **a**, CP_a, **b**, CP_b, **c**, CP_c, and **d**, CP_d. The competing RWGS reaction is shown in red.

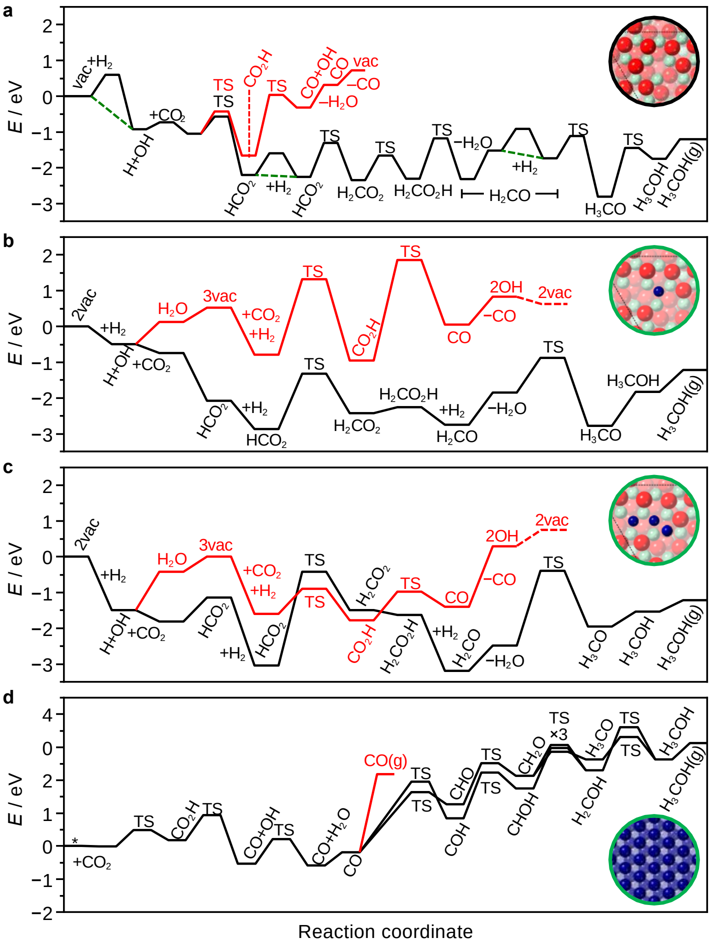

Supplementary Figure 15 | Energy profiles for CO_2 hydrogenation to methanol on **a**, unpromoted In_2O_3[1] **b**, DI_a, **c**, DI_b, and **d**, the Pd nanoparticle in DI_c modelled as Pd(111).[7,8] The competing RWGS reaction is shown in red.

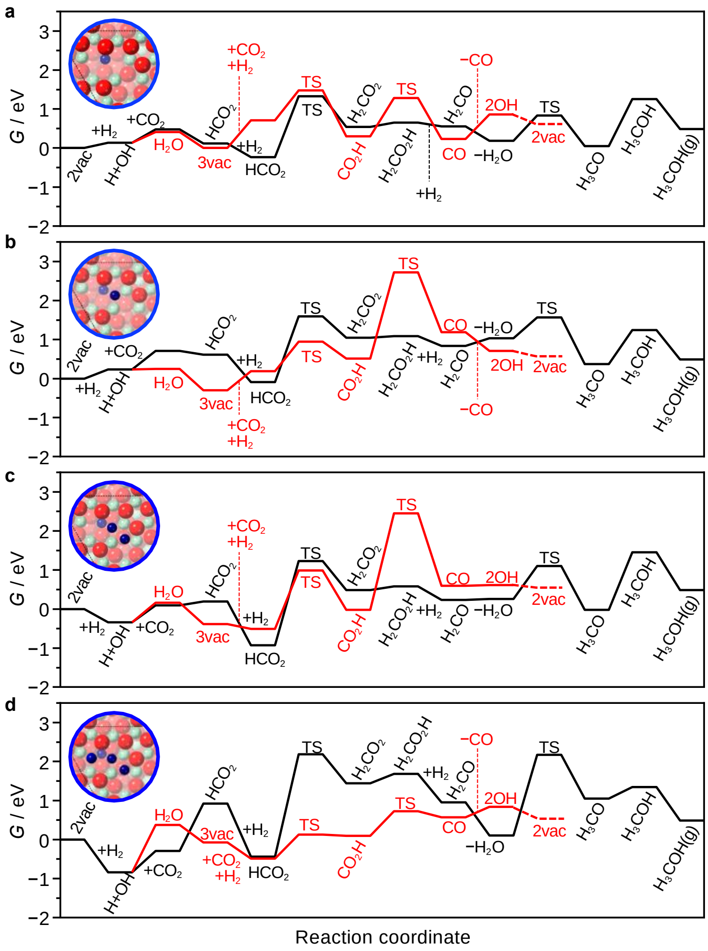

Supplementary Figure 16 | Gibbs energy profiles for CO_2 hydrogenation to methanol on **a**, CP_a, **b**, CP_b, **c**, CP_c, and **d**, CP_d. The competing RWGS reaction is shown in red.

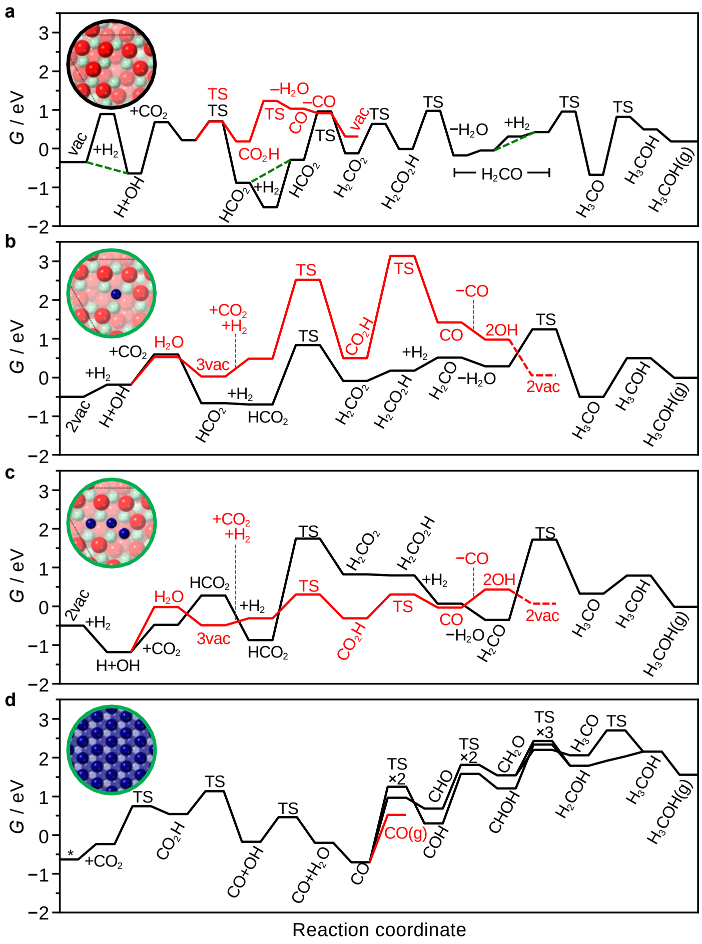

Supplementary Figure 17 | Gibbs energy profiles for CO_2 hydrogenation to methanol on **a**, unpromoted In_2O_3,[1] **b**, DI_a, **c**, DI_b, and **d**, the Pd nanoparticle in DI_c modelled as Pd(111).[7,8] The competing RWGS reaction is shown in red.

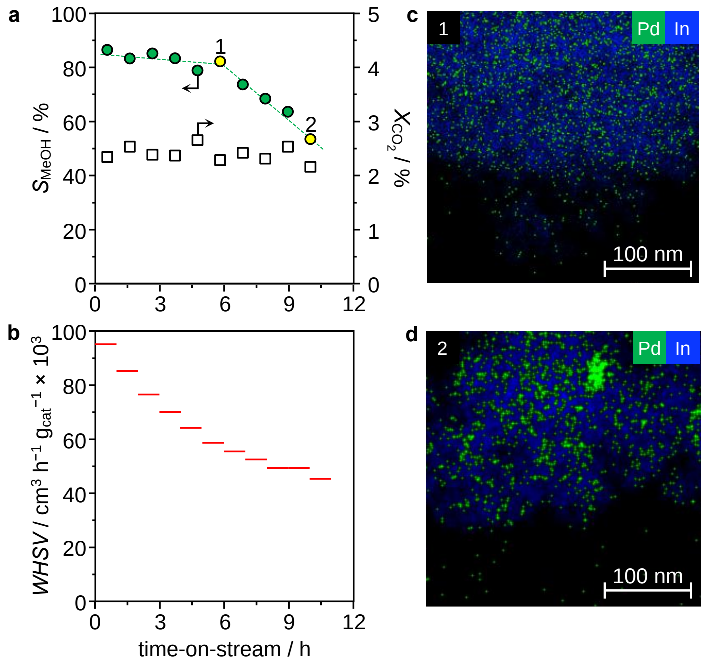

Supplementary Figure 18 | a, CO_2 conversion and methanol selectivity on the DI catalyst, tested at 553 K, H_2:CO_2 = 4, and variable *WHSV* as shown in **b**. Data are calculated from the average of 2 samples of the reactor outlet stream measured by GC and the carbon balance is associated with an error of 8%. The step-wise decrease of the residence time allowed to counterbalance catalyst deactivation, thus keeping the CO_2 conversion at *ca*. 2-3%. The selectivity reached at the end of the run is lower than for DI 74 h, since the reaction conditions were harsher in this experiment. **c**, and **d**, depict STEM-EDX maps of palladium and indium of the samples extracted from the reactor at the points indicated in **a**.

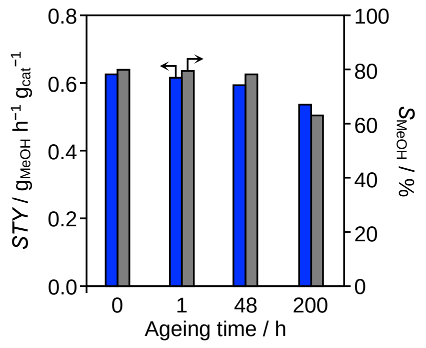

Supplementary Figure 19 | Methanol *STY* and selectivity on CP catalysts aged for 0-200 h. Reaction conditions: 553 K, 5 MPa, and H_2:CO_2 = 4, and *WHSV* = 24,000 cm^3 h^{-1} g$_{cat}$$^{-1}$.

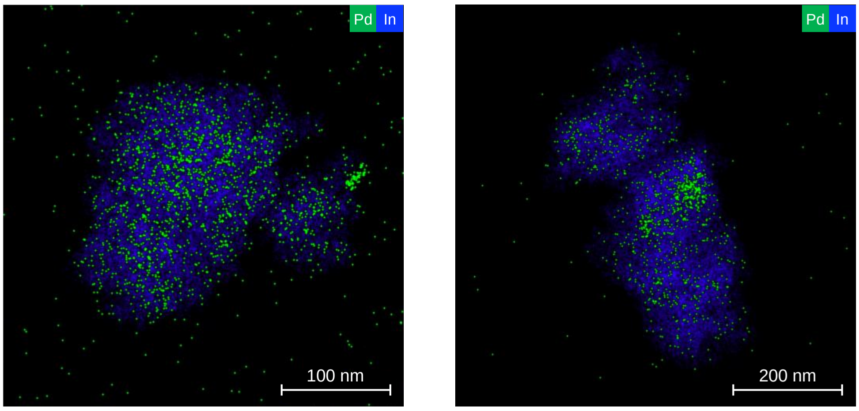

Supplementary Figure 20 | STEM-EDX maps of palladium and indium for the fresh CP catalyst aged for 200 h.

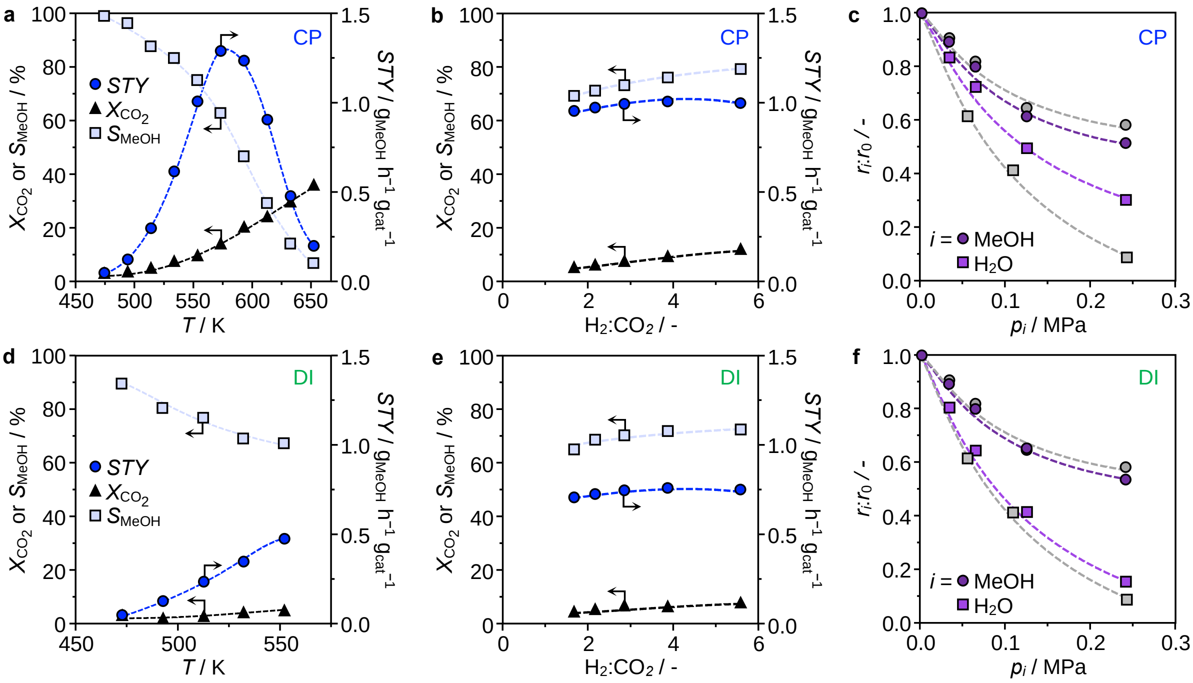

Supplementary Figure 21 | Dependence of CO_2 conversion, methanol selectivity, and methanol *STY* over the palladium-promoted In_2O_3 catalyst (0.75 wt.% Pd) obtained by CP on **a**, temperature and **b**, inlet $H_2:CO_2$ ratio, and **c**, dependence of the methanol formation rate on partial pressure of the products. Analogue experiments on the equilibrated palladium-promoted In_2O_3 catalyst (0.75 wt.% Pd) obtained by DI are shown in **d**, **e**, and **f**, respectively. The observed rate (r_i) was normalized to that measured under standard conditions (r_0), *i.e.*, $T = 553$ K, $P = 5$ MPa, $H_2:CO_2 = 4$, and $WHSV = 48{,}000\ cm^3_{STP}\ h^{-1}\ g_{cat}^{-1}$. Results previously published on pure In_2O_3[1] are added in grey to serve as a reference. When the $H_2:CO_2$ ratio was changed in the 1.8-5.6 range, higher CO_2 conversion and methanol selectivity were attained at higher relative concentrations of H_2, as expected, but due to the progressively lower amount of

CO_2 fed to the reactor, the methanol *STY* remained almost constant. Upon individually adding the reaction products to the feed mixture in the partial-pressure range of 0.05-0.25 MPa, a drop in methanol formation was observed, which was substantially less pronounced for water in the case of the CP catalyst with respect to pure In_2O_3 and the equilibrated DI sample.

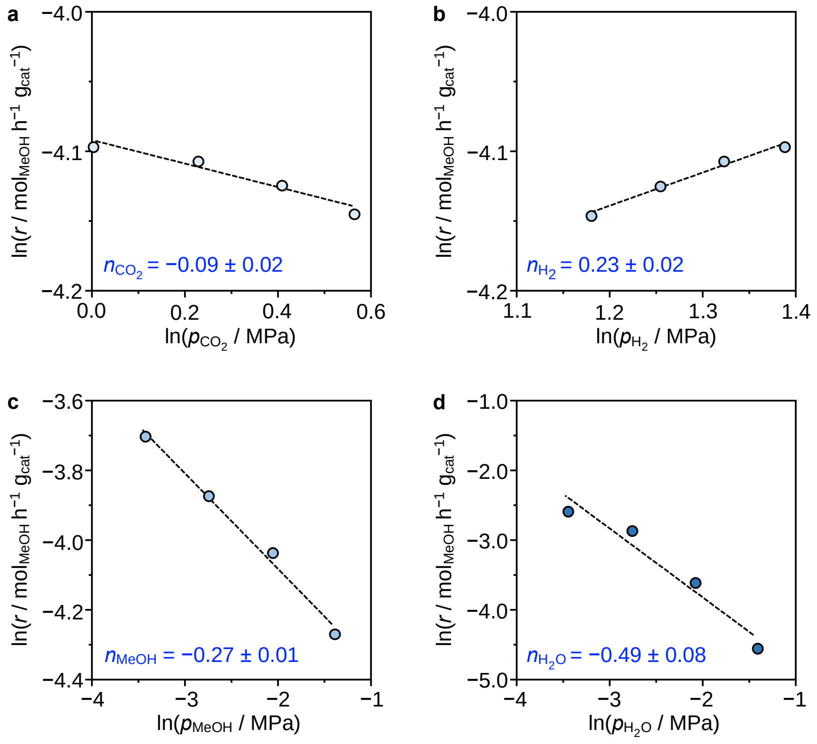

Supplementary Figure 22 | Regression of the data shown in **Supplementary Figures 8b-c** to extract the apparent reaction orders (n_i) of species *i* for **a**, CO_2, **b**, H_2, **c**, MeOH, and **d**, H_2O in CO_2 hydrogenation to methanol over the CP catalyst. As only one decimal is considered significant, the n_i values in the main manuscript are rounded accordingly. Reaction conditions: T = 553 K, P = 5 MPa, H_2:CO_2 = 4, and *WHSV* = 48,000 cm^3_{STP} h^{-1} g_{cat}^{-1}.

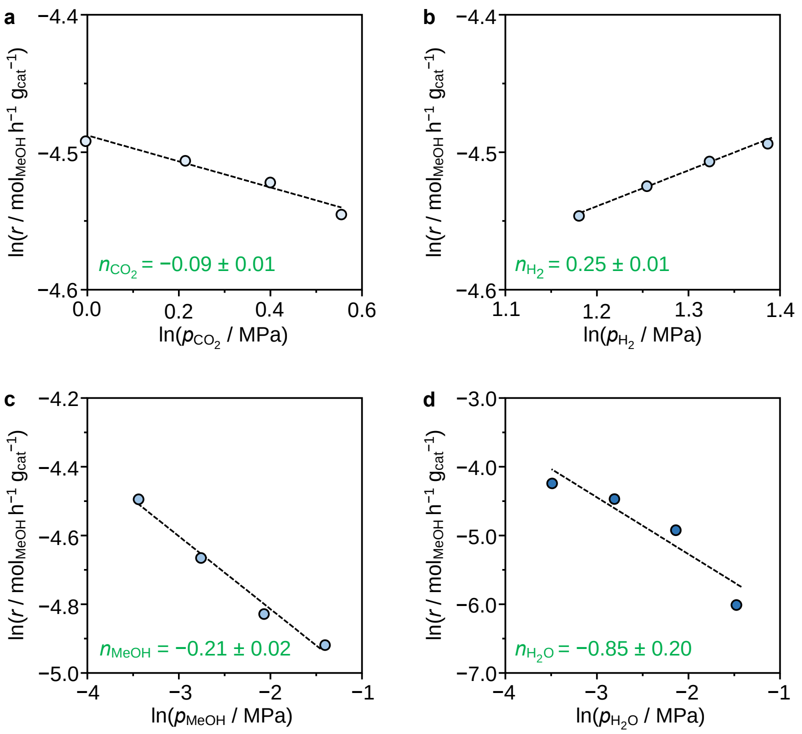

Supplementary Figure 23 | Regression of the data presented in **Supplementary Figures 8e-f** to extract the apparent reaction orders (n_i) of species i for **a**, CO_2, **b**, H_2, **c**, MeOH, and **d**, H_2O in CO_2 hydrogenation to methanol over the DI catalyst. As only one decimal is considered significant, the n_i values in the main manuscript are rounded accordingly. Reaction conditions: T = 553 K, P = 5 MPa, H_2:CO_2 = 4, and $WHSV$ = 48,000 cm^3_{STP} h^{-1} g_{cat}^{-1}.

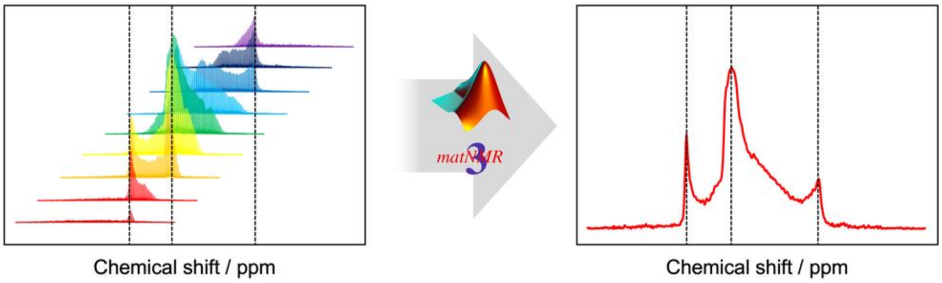

Supplementary Figure 24 | Schematic representation of the ^{115}In NMR data processing to generate one composite spectrum from individually acquired sub-spectra.

Supplementary Tables

Supplementary Table 1 | Synthetic approach, basic characterization data, and first appearance in the main manuscript for all the materials discussed. Key samples are shaded in grey.

Catalyst	Synthesis[a]	Nominal loading[b] [wt.%]	Measured loading[b,c] [wt.%]	V_{pore}[d] [cm^3 g^{-1}]	S_{BET}[d] [m^2 g^{-1}]	Relevant figure
In_2O_3	P	-	-	0.37	125	1a,b
**Pd**-In_2O_3	CP	0.1	0.08	0.46	145	1a
**Pd**-In_2O_3	CP	0.25	0.31	0.40	147	1a
**Pd**-In_2O_3	CP	0.5	0.47	0.50	126	1a
**Pd**-In_2O_3	CP	0.75	0.74	0.51	174	1
**Pd**-In_2O_3	CP	1.0	0.96	0.55	130	1a
**Pd**-In_2O_3	CP	1.5	1.45	0.56	149	1a
**Pd**-In_2O_3	CP	3.5	3.36	0.53	158	1a
**Pd**-In_2O_3	CP	10	9.79	0.50	112	1a
**Pd**-In_2O_3	CP[e]	0.75	0.73	0.21	133	1b
**Pd**-In_2O_3	DI	0.25	0.25	0.36	127	1a
**Pd**-In_2O_3	DI	0.75	0.73	0.35	131	1a
**Pd**-In_2O_3	DI	3.5	3.43	0.26	113	1a
**Pd**-In_2O_3	SG	0.75	0.75	0.30	108	S1a
**Pd**-In_2O_3	WI	0.75	0.73	0.37	121	S1a
**Pd**-In_2O_3	SD	0.75	0.63	0.40	131	S1a
**Pd**-In	CR	0.75	0.71	0.10	34	S1a
TiO_2	-	-	-	0.15	59	S1b
**Pd**-TiO_2	DI	0.75	0.72	0.12	58	S1b
**Pd**-TiO_2	CP	0.75	0.68	0.09	24	S1b
**Ag**-In_2O_3	CP	0.75	0.71	0.40	146	S1c
**Au**-In_2O_3	CP	0.75	0.68	0.39	113	S1c
**Ru**-In_2O_3	CP	0.75	0.78	0.38	134	S1c
**Cu**-In_2O_3	CP	0.75	0.75	0.43	157	S1c
**Pt**-In_2O_3	CP	0.75	0.74	0.44	150	S1c
**Ir**-In_2O_3	CP	0.75	0.88	0.29	117	S1c

[a]P: precipitation, CP: coprecipitation, DI: dry impregnation, SG: sol-gel method, WI: wet impregnation, SD: spray deposition, CR: chemical reduction, [b]Refers to the metal (bold) added to In_2O_3, In, or TiO_2, [c]Determined by ICP-OES, [d]Determined by nitrogen sorption. [e]Aged for 200 h.

Supplementary Table 2 | Surface concentration of elements for selected catalysts as determined by XPS analysis.

Catalyst[a]	In [at.%]	Pd [at.%]	C [at.%]	O [at.%]
CP fresh	37.6	0.2	7.2	55.0
CP 1 h[b]	39.9	0.4	6.6	53.2
CP 16 h[b]	39.1	0.5	7.3	53.2
DI 1 h[b]	37.8	1.1	8.7	52.6
DI 16 h[b]	33.8	0.6	6.8	58.8

[a]Nominal Pd loading = 0.75 wt.% (1.93 at.%), [b]Reaction conditions: $T = 553$ K, $P = 5$ MPa, H_2:CO_2 = 4, and $WHSV$ = 24,000 cm^3_{STP} h^{-1} g_{cat}^{-1}.

Supplementary Table 3 | Results of the XAS spectra fitting presented in **Supplementary Figure 7**. The uncertainty associated with the values is indicated in brackets.

Catalyst[a]	Nominal Pd loading [wt.%]	Scattering path	ΔE [eV]	Number of neighbors [-]	R_{eff} [Å]	R [Å]	Debye-Waller factor [Å^2]
DI fresh	0.75	Pd-O	6.6(1.6)	4.0(5)	2.0194	2.008(9)	0.0023(9)
DI 1 h[b]	0.75	Pd-Pd(In)	−1(2)	6.0(1.5)	2.7508	2.75(1)	0.011(2)
DI 16 h[b]	0.75	Pd-Pd(In)	−1(2)	7.2(2.0)	2.7508	2.76(1)	0.013(2)
CP fresh	0.75	Pd-O	7.7(9)	3.5(3)	2.0194	2.01(1)	0.0018(2)
		Pd-O-In	7.7(9)	6.7(1.5)	3.3478	3.35(2)	0.012(2)
CP 1 h[b]	0.75	Pd-Pd(In)	−5.4(3.0)	2.6(1.0)	2.7508	2.64(2)	0.008(2)
CP 16 h[b]	0.75	Pd-Pd(In)	−3.7(2.4)	4.2 (1.2)	2.7508	2.65(2)	0.010(2)
CP fresh	0.25	Pd-O	7.3(2.1)	3.5(7)	2.0194	2.01(1)	0.002(2)
		Pd-O-In	7.3(2.1)	7(3)	3.3478	3.30(2)	0.012(3)
CP fresh	3.5	Pd-O	6.8(1.7)	3.5(4)	2.0194	2.009(7)	0.0018(9)
CP 1 h[b]	3.5	Pd-Pd(In)	-0.4(2.7)	4(1)	2.7508	2.76(1)	0.007(2)
CR	50	Pd-Pd(In)	−0.1(5)	10.1(6)	2.7508	2.774(2)	0.0084(2)

[a]DI: dry impregnation, CP: coprecipitation, CR: chemical reduction. [b]Reaction conditions: $T = 553$ K, $P = 5$ Mpa, H_2:CO_2 = 4, and $WHSV$ = 24,000 cm^3_{STP} h^{-1} g_{cat}^{-1}.

Supplementary Table 4 | Calculated energies of substitution (E_{sub}), segregation (E_{seg}), and islanding (E_{isl}) associated with the replacement of In atoms in In_2O_3 by Pd atoms at surface and bulk lattice positions. Gaseous or solid palladium is used as the source of Pd atoms, whereby gas-phase palladium more closely represents the atomic dispersion of palladium in the precursor solutions used in the CP and DI syntheses. Regardless of the palladium source, vacancy formation, islanding, and segregation energies are unaffected since they are determined as a difference between initial and final states. E_{vac} is the energy required to remove the O atom in the center of the In_3O_5(O) ensemble depicted in **Figure 3g** and **Supplementary Figure 10**.

In substitution by Pd[a]	Structure	E_{sub}[b] [eV]	E'_{sub}[c] [eV]	E_{seg}[d] [eV atom^{-1}]	E_{isl}[e] [eV atom^{-1}]	E_{vac}[f] [eV]
none	S_0	–	–	–	–	–0.64
bulk	S_B	+3.56	–0.19	–	–	–0.63
1	S_1	+2.69	–1.06	–0.87	–	–0.58
2	S_2	+3.57	–0.61	–0.42	–	–2.07
3	S_3	+3.14	–0.18	+0.01	–	–1.50
1 and 2	S_4	+5.52	–1.98	–0.80	+0.07	–1.58
1 and 3	S_5	+6.37	–1.12	–0.37	+0.50	–1.51
2 and 3	S_6	+6.86	–0.63	–0.13	+0.75	–2.96
1, 2, and 3	S_7	+9.19	–2.04	–0.49	+0.38	–2.49

Numbers correspond to the labels in **Figure 3g**.

[b]Calculated from equation $In_{80}O_{120} + nPd_{(s)} + \frac{3n}{2} H_2O_{(g)} \rightarrow In_{79}O_{120}Pd_n + \frac{n}{2} In_2O_{3(bulk)} + \frac{3n}{2} H_{2(g)}$.

[c]Calculated from equation $In_{80}O_{120} + nPd_{(g)} + \frac{3n}{2} H_2O_{(g)} \rightarrow In_{79}O_{120}Pd_n + \frac{n}{2} In_2O_{3(bulk)} + \frac{3n}{2} H_{2(g)}$.

[d]Calculated for structure S_i *as* $\frac{1}{n} E_{sub}(S_i) - E_{sub}(S_B)$, where n is the number of In to Pd substitutions.

[e]Calculated for structure S_i as $E_{seg}(S_i) - E_{seg}(S_1)$.

[f]Calculated from the equation $In_{80-x}O_{120}Pd_x + H_{2(g)} \rightarrow In_{80-x}O_{119}Pd_x + H_2O_{(g)}$.

Supplementary Table 5 | Calculated adsorption energy of a cluster of *n* Pd atoms in the pocket of In_2O_3(111) shown in **Figure 3g** (E_{ads}), segregation energy averaged per Pd atom (E_{seg}), and islanding energy (E_{isl}). The comment about the source of palladium in **Supplementary Table 4** holds for this case too. E_{vac} is the energy required to remove the O atom in the center of the In_3O_5(O) ensemble (**Figure 3g** and **Supplementary Figure 10**).

Structure		Number of Pd atoms	E_{ads}[a] [eV]	E'_{ads}[b] [eV]	E_{seg}[c] [eV atom^{-1}]	E_{isl}[d] [eV atom^{-1}]	E_{vac}[e] [eV]
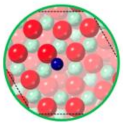	S_8	1	+1.98	–1.77	–0.71	–	–1.04
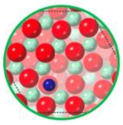	S_9	1	+2.24	–1.51	–0.45	–	–0.90
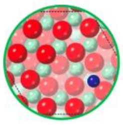	S_{10}	1	+2.32	–1.43	–0.37	–	–1.09
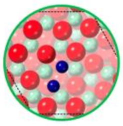	S_{11}	2	+3.75	–3.74	–0.81	–0.10	–1.37
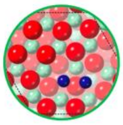	S_{12}	2	+4.13	–3.36	–0.62	+0.09	–1.22
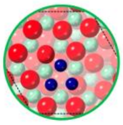	S_{13}	3	+4.53	–6.70	–1.17	–0.47	–0.95
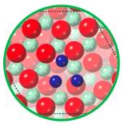	S_{14}	3	+5.39	–5.85	–0.89	–0.18	–1.61
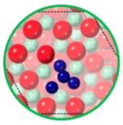	S_{15}	4	+5.87	–9.12	–1.22	–0.51	–0.94
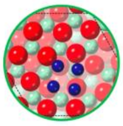	S_{16}	4	+6.12	–8.86	–1.15	–0.45	–0.69

[a]Calculated from equation $In_{80}O_{120} + nPd_{(s)} \rightarrow In_{80}O_{120}Pd_n$.

[b]Calculated from equation $In_{80}O_{120} + nPd_{(g)} \rightarrow In_{80}O_{120}Pd_n$.

[c]Calculated from equation $In_{79}Pd_{bulk}O_{120} + \frac{1}{2}In_2O_{3(bulk)} + \frac{3}{2}H_{2(g)} \rightarrow \frac{1}{n}In_{80}O_{120}Pd_n + \frac{n-1}{n}In_{80}O_{120} + \frac{3}{2}H_2O_{(g)}$.

[d]Calculated for structure S_i as $E_{seg}(S_i) - E_{seg}(S_8)$.

[e]Calculated from equation $In_{80}O_{120}Pd_x + H_{2(g)} \rightarrow In_{80}O_{119}Pd_x + H_2O_{(g)}$.

Supplementary Table 6 | Apparent activation energies obtained by microkinetics. The system names correspond to those on Figure 3. The number of exposed Pd atoms is indicated. $G_{a,MeOH}$ and $G_{a,RWGS}$ represent the apparent activation energies for methanol and CO production, respectively. The labels refer to the structures shown in **Figure 3g** in the main manuscript.

System	Exposed Pd atoms [–]	$G_{a,MeOH}$ [kJ mol^{-1}]	$G_{a,RWGS}$ [kJ mol^{-1}]
In_2O_3	–	137	166
CP_a	0	51	127
CP_b	1	81	245
CP_c	2	133	333
CP_d	3	405	159
DI_a	1	87	247
DI_b	3	353	154
Pd(111)	all	230	148

Supplementary Table 7 | Conditions applied in the catalytic tests.

Experiment[a]	Catalyst weight [mg]	Gas[b]	Flow rate[c] [cm^3_{STP} min^{-1}]					Temperature [K]
Standard activity test	100	CO_2	8					553
(**Figures 1a-b and S1a-c**)		H_2	32					
Activity test at X_{CO_2} = 3%	50[d]	CO_2	2.25, 10.5, or 19.5[e]					553
(**Figures 1c)**		H_2	9, 42, or 39[e]					
Determination of apparent	50[d]	CO_2	8					473-653[c,f]
activation energy		H_2	32					
(**Figure 4, S21a**)								
Determination of reaction	50[d]	CO_2	14	12	10	8	6	553
order with respect to CO_2		H_2	26	28	30	32	34	
and H_2 (**Figure S21b**)								
Determination of reaction	50[d]	CO_2	8	7.95	7.9	7.8	7.6	553
order with respect to MeOH		H_2	32	31.8	31.6	31.2	30.4	
(**Figure S21c**)		MeOH	0	0.25	0.5	1	2	
Determination of reaction	50[d]	CO_2	8	7.95	7.9	7.8	7.6	553
order with respect to H_2O		H_2	32	31.8	31.6	31.2	30.4	
(**Figure S21c**)		H_2O	0	0.25	0.5	1	2	
Extended activity test	50[d]	CO_2	8					553
(**Figure 6**)		H_2	32					
Extended test at const. X_{CO_2}	25[d]	CO_2	8[g]					553
(**Figure 6**)		H_2	32[g]					

[a]All experiments were carried out at 5 MPa. [b]20 mol% CH_4 in He was fed (2.5 cm^3_{STP} min^{-1}) to the effluent stream of the reactor as an internal standard. [c]Each experimental condition was applied for 3 h. [d]Diluted with 50 mg of TiO_2. [e]For In_2O_3, CP, or DI respectively. [f]The temperature was increased in steps of 20 K. [g]Varied as outlined in **Supplementary Figure 18**.

Supplementary Table 8 | Conditions applied in the temperature-programmed reduction and desorption analyses.

Analysis[a]	Gas	Pressure [MPa]	Flow rate [cm^3_{STP} min^{-1}]	Temperature [K]	Heating rate [K min^{-1}]	Dwell time [min]
**HP-H_2-TPR**						
Drying	Ar	0.1	100	303-393	5	60
Cooling	Ar	0.1	100	393-183	−10	10
Reduction with H_2	H_2(5 mol%)/Ar	5	50	183-1103	5	30
**H_2-TPR**						
Drying	Ar	0.1	100	303-393	5	60
Cooling	Ar	0.1	100	393-283	−10	10
Reduction with H_2	H_2(5 mol%)/Ar	0.1	20	283-1103	5	5
**CO_2-TPD**						
Reaction	CO_2(20 mol%)/H_2	5	100	303-553	5	60
Quenching	CO_2(20 mol%)/N_2	5	100	553-273	−90	0
CO_2 adsorption	CO_2(20 mol%)/N_2	5	100	273	-	15
Inert flushing	Ar	0.1	100	273	-	60
CO_2 d~~D~~esorption	Ar	0.1	50	273-1103	5	30
**CO-TPD**						
Drying	Ar	0.1	100	303-393	5	60
Cooling	Ar	0.1	100	393-283	−10	10
CO adsorption	CO(5 mol%)/N_2	0.1	100	283	-	30
Inert flush	Ar	0.1	100	283	-	60
CO desorption	Ar	0.1	20	283-1103	5	5

[a]100 mg of catalyst were loaded for each analysis, HP = high pressure.

Supplementary Table 9 | Theoretical Bader charge (q_B) and XPS Pd3d shifts relative to metallic Pd (E_{3d}) for the Pd atoms in the structures depicted in **Figure 3**.

Structure		Position	q_B [|e⁻|][a]	E_{3d} [eV][b]	Structure		Position	q_B [|e⁻|][a]	E_{3d} [eV][b]
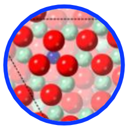	CP fresh	–	+1.3	+2.9	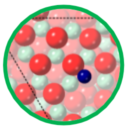	DI fresh	–	+0.1	–0.7
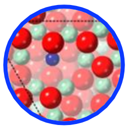	CP_a	–	+0.5	–0.2	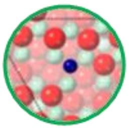	DI_a	–	–0.4	+0.5
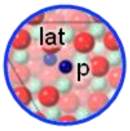	CP_b	lat<br>p	+0.6<br>–0.2	+0.3<br>–0.1	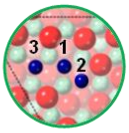	DI_b	1<br>2<br>3	–0.3<br>–0.3<br>–0.3	–0.1<br>–0.2<br>–0.2
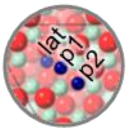	CP_c	lat<br>p1<br>p2	+0.7<br>–0.2<br>–0.1	+1.9<br>–0.4<br>–0.7	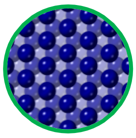	DI_c	–	–0.1	–0.3

[a]Bulk Pd: +0.1 |e⁻|; surface Pd: –0.1 |e⁻|; PdO: +0.9 |e⁻|; PdO_2: +1.4 |e⁻|.

[b]Surface Pd: –0.3 eV; PdO; +1.1 eV; PdO_2: +2.7 eV.

Supplementary References

1. Frei, M. S. *et al.* Mechanism and microkinetics of methanol synthesis *via* CO_2 hydrogenation on indium oxide. *J. Catal.* **361**, 313-321 (2018).
2. Abdala, P. M. *et al.* Scientific opportunities for heterogeneous catalysis research at the SuperXAS and SNBL beam lines. *Chimia* **66**, 699-705 (2012).
3. Ravel, B. & Newville, M. ATHENA, ARTEMIS, HEPHAESTUS: Data analysis for X-ray absorption spectroscopy using IFEFFIT. *J. Synchrotron Radiat.* **12**, 537-541 (2005).
4. van Beek, J. D. matNMR: A flexible toolbox for processing, analyzing and visualizing magnetic resonance data in Matlab. *J. Magn. Reson.* **187**, 19-26 (2007).
5. Tauster, S. Strong metal-support interactions. *Acc. Chem. Res.* **20**, 389-394 (1987).
6. Snider, J. L. *et al.* Revealing the synergy between oxide and alloy phases on the performance of bimetallic In–Pd catalysts for CO_2 hydrogenation to methanol. *ACS Catal.* **9**, 3399-3412 (2019).
7. García-Muelas, R.; Li, Q. & López, N. Density functional theory comparison of methanol decomposition and reverse reactions on metal surfaces. *ACS Catal.* **5**, 1027-1036 (2015).
8. Li, Q.; García-Muelas, R. & López, N. Microkinetics of alcohol reforming for H_2 production from a fair density functional theory database. *Nat. Commun.* **9**, 526 (2018).
