# Supplementary material for: Atomic-scale engineering of indium oxide promotion by palladium for methanol production via CO2 hydrogenation
Source: Nat Commun. 2019 Jul 29;10:3377. doi: 10.1038/s41467-019-11349-9 (PMC6662860; doi:10.1038/s41467-019-11349-9)
Supplement: Supplementary file 2 — Peer Review [file 41467_2019_11349_MOESM2_ESM.pdf]

Editorial Note: this manuscript has been previously reviewed at another journal that is not operating a transparent peer review scheme. This document only contains reviewer comments and rebuttal letters for versions considered at *Nature Communications.*

REVIEWERS' COMMENTS:

Reviewer #1 (Remarks to the Author):

The revision and responses by the authors addressed my /comments/concerns satisfactorily. I would recommend publishing this contribution in Nature Communication.

Reviewer #2 (Remarks to the Author):

The authors of the submitted manuscript have extensively revised their original submission based on the provided feedback from reviewers. The data is clearly explained and generally supportive of the conclusions, and the additional discussions that have been added/edited are thorough. I believe this represents a good addition to the field.
